# Concordant and discordant DNA methylation signatures of aging in human blood and brain

**DOI:** 10.1186/s13072-015-0011-y

**Published:** 2015-05-09

**Authors:** Pau Farré, Meaghan J Jones, Michael J Meaney, Eldon Emberly, Gustavo Turecki, Michael S Kobor

**Affiliations:** Department of Physics, Simon Fraser University, 8888 University Drive, Burnaby, BC V5A 1S6 Canada; Centre for Molecular Medicine and Therapeutics, Child & Family Research Institute, 950 W 28th ave, Vancouver, BC V5Z4H4 Canada; Department of Medical Genetics, University of British Columbia, 950 W 28th ave, Vancouver, BC V5Z4H4 Canada; Ludmer Centre for Neuroinformatics and Mental Health, Douglas Mental Health University Institute, McGill University, 6875 Boulevard Lasalle, Verdun, QC H4H 1R3 Canada; Singapore Institute for Clinical Sciences, 30 Medical Drive, Singapore, 117609 Singapore; Canadian Institute for Advanced Research, Toronto, ON Canada; Department of Psychiatry, McGill University, 6875 Boulevard Lasalle, Verdun, QC H4H 1R3 Canada

**Keywords:** DNA methylation, Principal component analysis, Aging, Brain, Blood, Epigenetics

## Abstract

**Background:**

DNA methylation is an epigenetic mark that balances plasticity with stability. While DNA methylation exhibits tissue specificity, it can also vary with age and potentially environmental exposures. In studies of DNA methylation, samples from specific tissues, especially brain, are frequently limited and so surrogate tissues are often used. As yet, we do not fully understand how DNA methylation profiles of these surrogate tissues relate to the profiles of the central tissue of interest.

**Results:**

We have adapted principal component analysis to analyze data from the Illumina 450K Human Methylation array using a set of 17 individuals with 3 brain regions and whole blood. All of the top five principal components in our analysis were associated with a variable of interest: principal component 1 (PC1) differentiated brain from blood, PCs 2 and 3 were representative of tissue composition within brain and blood, respectively, and PCs 4 and 5 were associated with age of the individual (PC4 in brain and PC5 in both brain and blood). We validated our age-related PCs in four independent sample sets, including additional brain and blood samples and liver and buccal cells. Gene ontology analysis of all five PCs showed enrichment for processes that inform on the functions of each PC.

**Conclusions:**

Principal component analysis (PCA) allows simultaneous and independent analysis of tissue composition and other phenotypes of interest. We discovered an epigenetic signature of age that is not associated with cell type composition and required no correction for cellular heterogeneity.

**Electronic supplementary material:**

The online version of this article (doi:10.1186/s13072-015-0011-y) contains supplementary material, which is available to authorized users.

## Background

Epigenetics refers to modifications to DNA and chromatin that regulate transcription without alteration of the genetic code. The best-studied epigenetic mark is DNA methylation, first defined as the addition of a methyl group to a cytosine residue, most frequently in the context of CG dinucleotides. CpGs are not uniformly distributed in the genome and tend to be enriched in CpG islands [1,2]. Most promoters in the genome have an associated CpG island, and DNA methylation levels at these promoter-associated islands associate with gene expression levels [2]. CpG density is commonly classed into four categories: high-density CpG island (HC), intermediate density CpG island (IC), intermediate density island shore (ICshore, meaning intermediate density regions found flanking HC regions), and low-density CpG island (LC) [2,3]. This CpG density is related to both DNA methylation level and variability [1,2]. While the common form of DNA methylation described above has been the most extensively studied, many other related modifications have recently emerged. Chief among them is 5-hydroxymethylcytosine, which exists as the oxidized form of the canonical 5-methylcytosine mark of DNA methylation. Catalyzed by the ten-eleven translocation (TET) family of enzymes, DNA hydroxymethylation is thought to exist as an intermediate in the process of active DNA demethylation, although its exact functional role remains enigmatic [4,5]. DNA hydroxymethylation in the mammalian brain is typically 5 to 10 times higher than any other tissue, and recent evidence has suggested that it plays an important role in normal brain function [6-9]. Importantly, most sodium bisulfite-based methods of measurement of DNA methylation cannot distinguish between the different kinds of modifications.

Epigenetics in general and DNA methylation in particular are associated with cell fate and differentiation. Landscapes of DNA methylation are highly divergent between cell types, with cells from similar lineages showing more similar DNA methylation profiles [10,11]. In the context of DNA methylation, the main drivers of tissue-specific patterns are located in areas of low CpG density outside of islands and shores, and so differences between tissues are often found in discrete locations in the genome [12,13].

Studies of the differences in DNA methylation between cell types in humans pose two difficulties. First, since in many cases the tissue of interest for a particular condition may not be available, surrogate tissues are often employed. In particular, studies of the brain necessarily require the use of postmortem tissue, tissue from surgical resection, or a surrogate tissue. Postmortem tissue has been useful in many studies, but in general for large-scale studies, only surrogate tissue is available [14,15]. In these cases, researchers examine accessible peripheral tissues like buccal cells or blood to examine associations with phenotypes that are assumed to be manifested in central tissues. Since different tissues show distinct epigenetic patterns, it is important to compare the DNA methylomes of peripheral and central tissues. One area of particular interest is determining whether the variation between individuals in a peripheral tissue resembles that in central tissues. Second, when comparing a specific tissue across individuals, differences in the cellular composition of the tissue sample can greatly affect DNA methylation pattern differences between the individuals [16]. This problem can be corrected by either measuring the tissue composition of the sample, by using the DNA methylation profiles themselves to predict the underlying cell composition of the tissue sample, or by using methods that correct for underlying cell composition without the actual measurements [17-20]. It is therefore important to develop a method that can assess the concordance between a surrogate tissue and the central tissue it represents while simultaneously controlling for cell composition differences in both tissues.

Organismic aging is a major component of epigenetic variation. Epigenetic aging consists principally of two distinct types of changes, termed ‘epigenetic drift’ and the ‘epigenetic clock’ [21-24]. Epigenetic drift refers to an increase in inter-individual variability with age, while the epigenetic clock refers to observations that specific sites in the genome show DNA methylation changes that are highly correlated with age across individuals. All tissues examined show an overall increase in DNA methylation with age, with some sites showing loss in methylation [14,25-32]. These changes tend to occur at genes related to developmental processes. Sites that gain DNA methylation with age tend to be located in islands, while sites that lose methylation are less likely to be found in islands, indicating a trend towards median levels of DNA methylation with age [11,14].

Studies of DNA methylation in cohorts present additional challenges. Sample size is often limited when interrogating precious primary human material from central organs [33]. This issue results in statistical challenges due to multiple testing controls when applying current high dimensional methodologies to measure the methylation status of a large number of CpGs. While correlational analysis with *P* values adjusted for multiple testing is commonly used, other methods are emerging that identify a reduced number of common patterns of variation across probes, lessening the impact of multiple statistical tests [34]. In addition, since CpGs tend to show highly correlated methylation profiles, especially CpGs situated in proximity, statistical approaches that assume probe independence are not ideally suited to the study of DNA methylation. In contrast, principal component analysis (PCA) is based on the cognizance that CpGs in an individual often share common patterns of DNA methylation [10,20,35]. PCA is a technique that identifies correlations among data points within a large multidimensional data set and is useful at reducing the dimensionality of the data. A given principal component (PC) describes a particular pattern of DNA methylation across samples. Each sample in the data set is assigned a score for each principal component, indicating the relative contribution of each PC-related pattern to the sample’s overall pattern. Each PC is also linearly independent from the others and accounts for a particular amount of variance within the data. PCA has often been used to identify batch effects in DNA methylation data, but has recently begun to be appreciated for its potential in broader and more biological aspects of epigenetic analysis [10,36-38].

We used a PCA approach to compare DNA methylation in brain and blood samples from 17 individuals. This matched design allows for rigorous assessment of DNA methylation irrespective of inter-individual differences in environment or genetic background. Given the large number of tests and the relatively small sample size, PCA, which allows for the identification of dominant patterns of variation in methylation between tissues and also across individuals within a tissue, was an appropriate choice. We found that PCA robustly identified patterns of DNA methylation associated with known traits even in this small cohort, two of which we validated in independent larger cohorts. The results presented here identify a PCA-based age predictor, as well as specific genomic locations where DNA methylation is more or less variable in brain and blood tissue.

## Results and discussion

Blood and brain samples were obtained from the Douglas-Bell Canada Brain Bank. A total of 17 participants were included in the study, ranging from 15 to 87 years of age, with 4 females and 13 males. Three cortical regions (Broadmann area 10 (BA10), prefrontal cortex; Broadmann area 7 (BA7), parietal cortex; and Broadmann area 20 (BA20) temporal cortex) were dissected from postmortem brain as described previously [39], and whole blood was collected postmortem from each subject by venipuncture. We used the Infinium Human Methylation 450K array to determine the genomic DNA methylation profiles of the three brain regions and matching peripheral whole blood. It is important to note that this technique, as currently applied, does not distinguish between DNA methylation and DNA hydroxymethylation, so our reports of DNA methylation in brain particularly are a composite of both marks. We obtained all 4 tissues of interest for 15 of the 17 participants; BA20 was missing from one participant and whole blood sample from another. We removed poorly performing probes, including those that overlapped with SNPs or hybridized to multiple locations in the genome and those located on the X and Y chromosomes, resulting in a total of 408,576 probes [3]. We applied PCA to this dataset to identify the major patterns of variation in DNA methylation.

### The majority of variation in DNA methylation was accounted for by tissue differences, cellular heterogeneity within a tissue, and subject age

**Figure 1:**
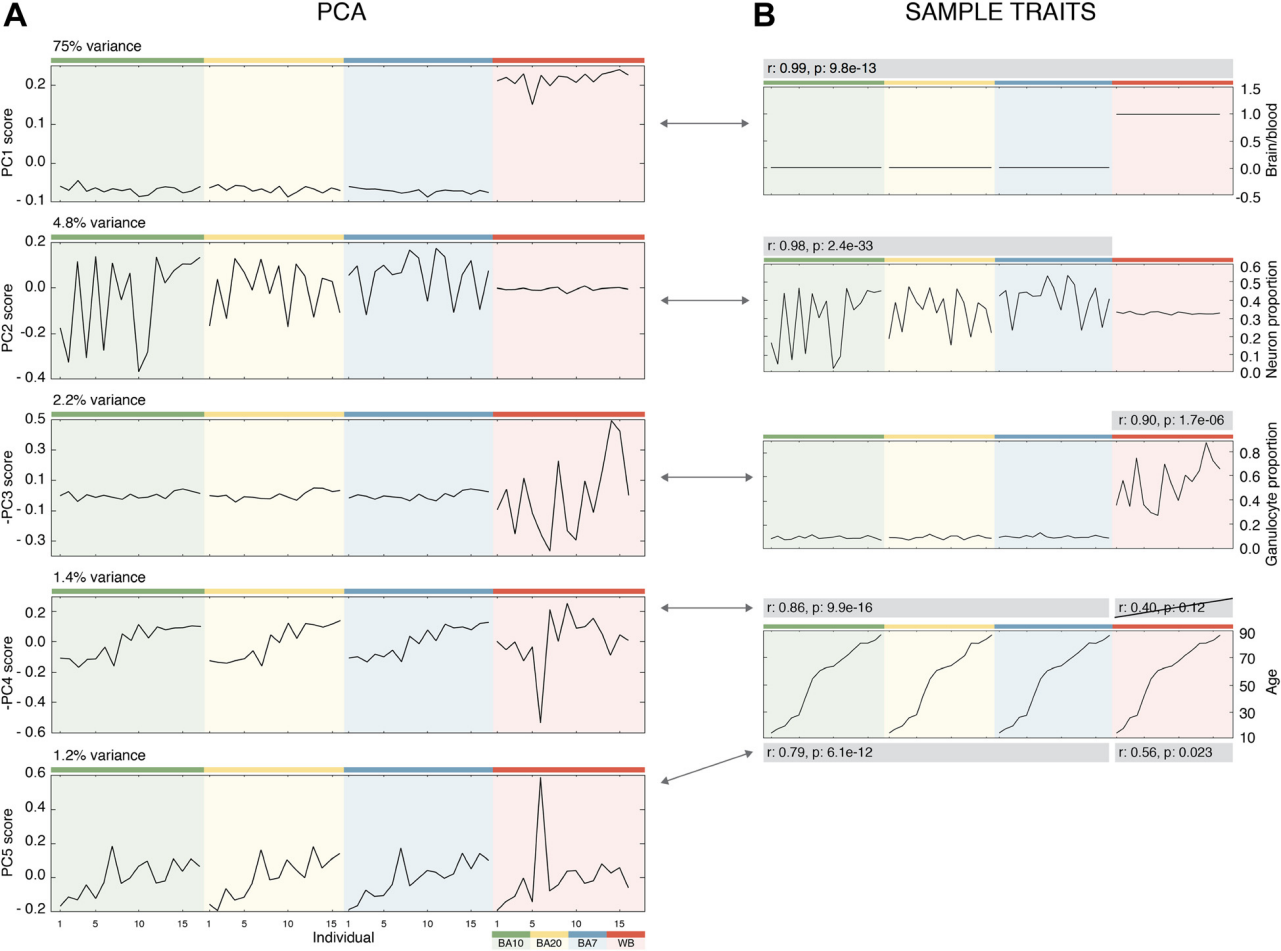
The first five principal components were associated with specific biological factors. **(A)** First five PCs and their associated variance. Samples were sorted by their tissue of origin (background color) and ordered by increasing age of the individual for each tissue. **(B)** Sample variables that correlated with the first five PC patterns. Correlations are shown in the gray boxes. Arrows connect PCs with the variables with which they are correlated. BA10, Broadmann area 10; BA20, Broadmann area 20, BA7, Broadmann area 7; PC, principal component; WB, whole blood.

We first identified the distinct principal components and determined their contribution to the total variance in our dataset (Additional file 1: Figure S1A). The first 13 PCs accounted for more than 90% of the variance in the data (Additional file 1: Figure S1A). Patterns of DNA methylation across samples for each PC were quite distinct, as illustrated for the top five PCs (Figure 1A). Each panel was first ordered by tissue and second by increasing subject age within each tissue. Both positive and negative correlations with PCs indicate the same type of relationship, since the sign of a particular PC score is arbitrary.

The majority of the variation (75%) was accounted for by PC1, which clearly separated brain from blood tissue, suggesting that the dominant difference in DNA methylation across all samples was the difference between blood and brain tissues (Figure 1A,B). Previous studies reveal that between-tissue differences are the main predictors of DNA methylation variability [10,20,40]. This PC likely included a contribution of hydroxymethylated sites, since hydroxymethylation is significantly higher in the brain than in the blood [8,9].

We also identified PCs with variability apparent only in brain tissue (PC2 in Figure 1A), in blood only (PC3 in Figure 1A), or across all tissues (PC4 and PC5 in Figure 1A, PC6 in Additional file 1: Figure S1D). The observation that PCs 2 and 3 both showed variability in only one of the two tissues raised the possibility that cellular composition within each tissue was underlying the pattern of variability observed. To test this hypothesis, we used cellular composition prediction algorithms on our DNA methylation data for each sample. This analysis resulted in predicted proportions of white blood cell types for the blood samples and a neuron/glia proportion for the brain samples [41,42]. Using these predicted proportions, we found that PC2, which was variable in all three brain regions but not blood, was highly correlated with the predicted proportion of neurons in the sample (*r* = 0.98, *P* = 2.4E−33, Figure 1B, scatter plot in Additional file 2: Figure S2). In contrast, PC3, which was variable in blood, but not brain, was highly correlated with the predicted proportion of granulocytes in the whole blood sample, but no other white blood cell types (*r* = −0.90, *P* = 1.7E−6, Figure 1B, scatter plot in Additional file 2: Figure S2). This finding was consistent with granulocytes being the predominant white blood cell type and thus likely contributing the majority of signal to a DNA methylation profile in whole blood. Collectively, these data showed that after tissue identity, cellular heterogeneity within a tissue was the major predictor of variation in DNA methylation.

When the samples were plotted by age (Figure 1), a trend was observed within the brain for PC4 and both brain and blood for PC5 (Figure 1A). We thus correlated the DNA methylation profiles for each PC with the age of the individuals. PC4 showed a statistically significant correlation with age for the three brain regions, but not blood. In contrast, there was a significant correlation for PC5 that included both brain and blood (Figure 1B, scatter plots in Additional file 2: Figure S2). These data suggest two distinct DNA methylation signatures of aging, one specific for the brain and the other encompassing both brain and blood. Interestingly, we also observed an increase in the mean level of DNA methylation with age in the brain, but not in blood samples (*r* = 0.71, *P* = 6.3E−9, Additional file 1: Figure S1B).

We then determined the contribution of individual CpG probes to a particular pattern of methylation variation across tissues and/or individuals by calculating the projection of each CpG site to each PC. A projection indicates either a positive or a negative contribution of the given PC pattern to the methylation profile observed at each CpG probe. Visualization of the distribution of CpG projections on PC1, which differentiates brain tissues from blood, clearly highlighted probes that had either a positive contribution (more methylated in the blood compared to the brain) or a negative contribution (more methylated in the brain than in the blood) (Additional file 3: Figure S3). Selecting probes by their projection score compared to standard deviation of all projections revealed that larger scores were reflective of greater similarities of the pattern of the individual CpG site to the overall pattern of PC1 (Additional file 3: Figure S3). For example, the two probes on the bottom left and bottom right, belonging to the group of ≷ ± 3*σ*, respectively, showed sharp transitions in DNA methylation between blood and brain, akin to PC1 (Additional file 2: Figures S2, Additional file 3: Figure S3 and 1A). This analysis illustrated that PC projections could be used as a means to filter the data to highlight specific traits in the classification approach used below. Nevertheless, it is important to note that, although greater projections imply greater associations with a PC, projections are not a measurement of statistical confidence. Instead, they simply quantify in which CpG sites the corresponding pattern of variation is the strongest. Mapping projection values to a confidence test would suppose a bias, as the PCs are obtained from the data, and, by definition, they are the most dominant patterns of variation [43].

### Variability of DNA methylation between brain and blood was moderately concordant

Having identified biological variables that were correlated with nearly 85% of the variability in our DNA methylation data, we next addressed the degree to which methylation varied across individuals, and whether this variability was consistent across tissues. Examining the distribution of M-value variance across all the samples of each tissue (Additional file 4: Figure S4A), we found that blood is significantly more variable than any of the brain tissues (Kolmogorov-Smirnov (KS)-test of blood vs brain regions >0.18; KS-test between brain regions <0.12; percentage of total variance in each tissue: BA10 26.5%, BA20 19.3%, BA7 17.7%, whole blood (WB) 36.4%). However, given the results in the previous section and previously published findings, it is likely that part of the inter-tissue differences in variance were due to differences in cell composition between tissues [16,20,44]. To account for this, we subtracted the contribution of PC2 (neuronal proportion) and PC3 (granulocyte proportion) from all samples and repeated the analysis (Additional file 4: Figure S4B). After removing this variance due to inter-individual differences in cellular composition, we found that the variances of different brain regions became notably homogenized (KS-test between brain regions <0.04) while the difference between brain and blood increased (KS-test of blood vs brain regions >0.27). We thus concluded that blood was significantly more variable han brain regions (percentage of total variance in each tissue: BA10 20.8%, BA20 21.5%, BA7 20.3%, WB 37.3%) and that inter-individual differences in cellular composition were an important contributor to variance in uncorrected data.

Our approach of using matched tissue samples allowed us to determine the fraction of PCs for which the observed pattern of variation was common in blood and brain. This analysis compared the similarity in the DNA methylation patterns between blood and cortical brain cells, with the caveat that brain DNA methylation includes both DNA methylation and DNA hydroxymethylation. Since PC1 to PC3 were identified as tissue- and cell-type-associated PCs, they would not be informative in determining concordance across tissues. For this analysis, we instead used the remaining PCs after PC3. We first averaged the three different brain tissue PCs together since their patterns of methylation are overall very similar. We next selected PCs for which the amount of variation in each tissue was of comparable magnitude: $\left|{\sigma}_{\mathrm{blood}}^2-{\sigma}_{\mathrm{brain}}^2\right|<\frac{1}{2}\left({\sigma}_{\mathrm{blood}}^2+{\sigma}_{\mathrm{brain}}^2\right).$ Finally, we selected the PCs showing a correlation *P* value of <0.01 between DNA methylation patterns of the brain and blood. The PCs that followed these criteria (19 PCs, 37.2% of variation after PC3) represented patterns of inter-individual variation that were common between blood and brain tissues (Additional file 5: Figure S5). The first eight of the PCs identified by these criteria were positively correlated between blood and brain and captured 74.5% of the variation, whereas the remaining PCs were negatively correlated between blood and brain and captured the remaining 25.5%. Thus, overall 37.2% of the non-tissue-specific variation was highly correlated between blood and brain, 74.5% of which is positively correlated and 25.5% is negatively correlated (Additional file 6: Figure S6). It is tempting to speculate that a portion of this shared variation between blood and brain might represent shared tissue differences, genetic impacts, or environmental exposures. The negatively correlated variation implies that high DNA methylation values in one tissue are associated with low methylation in the other. These sites may be those with important functions in either brain or blood, where they are highly expressed and low methylated in one tissue and not expressed and highly methylated in the other. These hypotheses based on our analysis of general patterns of DNA methylation will, of course, need to be tested, particularly to determine the possible contribution of hydroxymethylation to these differences and similarities in variation.

### Age-related PCs were more easily detected in the brain than in blood

We next performed PCA on each of our brain and blood tissues separately to see if the correlations with variables of interest would persist in a smaller dataset. All the brain tissues show a first PC that correlates with the neuron composition of the samples (*P* values <2E−11, Additional file 7: Figure S7A,B,C), followed by a second PC that correlates with age (*P* values <8E−6, Additional file 7: Figure S7E,F,G). Since both PC4 and PC5 in the full dataset showed a correlation with age in the brain tissue, all of these probes were found to strongly overlap with the probes identified with the age PCs in the brain tissues only. Interestingly, a PCA on the 16 blood samples revealed that the first two PCs both correlated with blood cell composition (first PC *P* value 2E−4, Additional file 7: Figure S7D; second PC *P* value 1E−2, Additional file 7: Figure S7H), but we were unable to identify a PC strongly correlated with age. This finding implies that the epigenetic pattern associated with age is not as strong in blood as it is in brain. Thus, PC5 in the full dataset, which shows an age-correlated pattern of methylation in blood as well as brain would not have been observed in blood alone, and was apparent because the presence of the pattern on brain reinforced power across samples.

We next sought to evaluate the sample size for a cohort required to detect an age-correlated PC in blood. We used an independent, published blood dataset (GSE40279) consisting of 656 individuals with an age range of 19 to 101 years [27]. First, we calculated the cell composition of the samples and subtracted the associated variance [41]. Then, we randomly subsampled the data into datasets with a smaller number of individuals. We performed PCA on each of the subsampled datasets and reported the percentage of times that we found an age-correlating PC for a given sample size. We found that in datasets consisting of 16 blood samples such as ours, the likelihood of finding a PC that correlates with age (*P* value <0.01) is approximately 41%. The chance of detecting an age-related PC in blood improves to >60% when there are more than 22 samples (Additional file 8: Figure S8). Although blood DNA methylation showed more variation than brain DNA methylation, a greater number of samples are needed to identify an age-related pattern of methylation, whereas in brain, a small sample size seems effective at identifying such a correlation.

### Age-associated PCs were replicated in independent datasets

**Figure 2:**
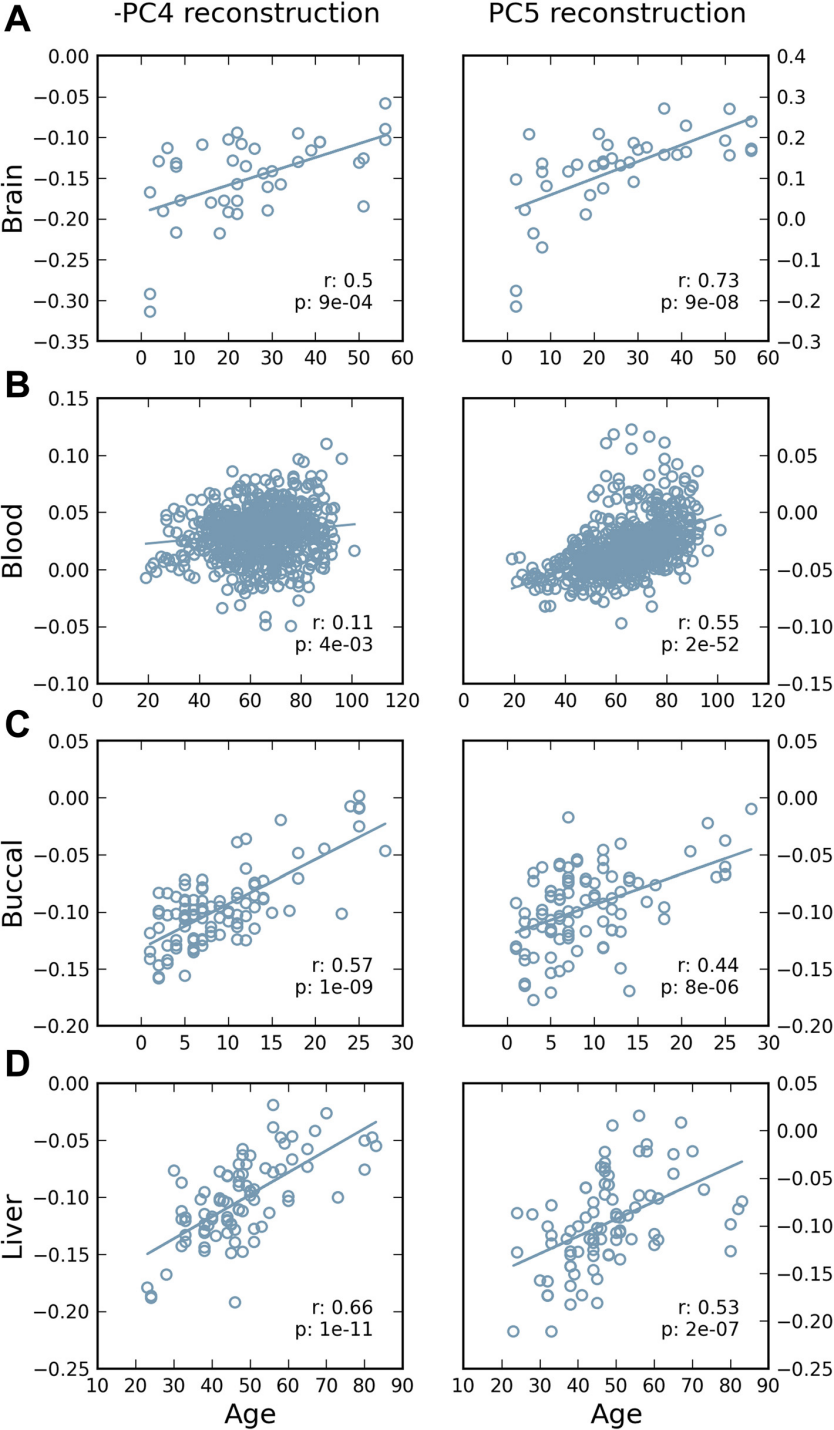
Age-associated PCs validated in independent datasets. The projections of each CpG site onto PC4 and PC5 were used to reconstruct these PCs in **(A)** 40 brain samples, **(B)** 656 blood samples, **(C)** 96 buccal swabs, and **(D)** 85 liver samples. *r* values and *P* values for each correlation are shown. PC, principal component.

We took advantage of published datasets to evaluate the reproducibility of our findings of tissue-concordant and tissue-discordant DNA methylation signatures of aging. We used the projections of each probe for the two PCs (PC4 and PC5) associated with aging and reconstructed these PCs on the larger published cohort. Beginning with data from brain (*n* = 40; age range: 2 to 56 years, GSE53162, [45]), we found a high correlation with age for reconstructed PC4 and PC5, similar to our brain data (Figure 2A). We next performed the same analysis on blood data (*n* = 656; age range 19 to 101 years, GSE40279, [27]) where we predicted that only the reconstructed PC5 would be correlated with age, since the original PC5 was correlated with age in both brain and blood tissue, while the original PC4 was correlated only in brain. Indeed, while reconstructed PC4 had a poor correlation coefficient with age (correlation: 0.11, *P* value 3.6E−3), reconstructed PC5 had very strong correlation that was highly significant (correlation: 0.55, *P* value 1.81E−52, Figure 2B). To more broadly investigate the tissue specificity of our aging signature, we performed the same reconstruction on two other data sets, from buccal epithelial cells (BEC) (*n* = 96; age range 1 to 28 years, GSE50759, [46]), and liver (*n* = 85; age range 23 to 83 years, GSE48325, [47]). Despite the younger and more limited age range of the BEC cohort, both data sets showed high correlation between both reconstructed PC4 and PC5 and age (Figure 2 for details, correlations, and *P* values). Collectively, these data suggested that at least two independent DNA methylation signatures of aging exist, one of which (PC5) is shared across all tissues examined and the other (PC4) which is found in all except blood. Inherently, these data also provided strong evidence for replication of age-related DNA methylation signatures between diverse datasets from different cohorts and laboratories.

### Hierarchical clustering of data using principal components revealed further relationships between samples

**Figure 3:**
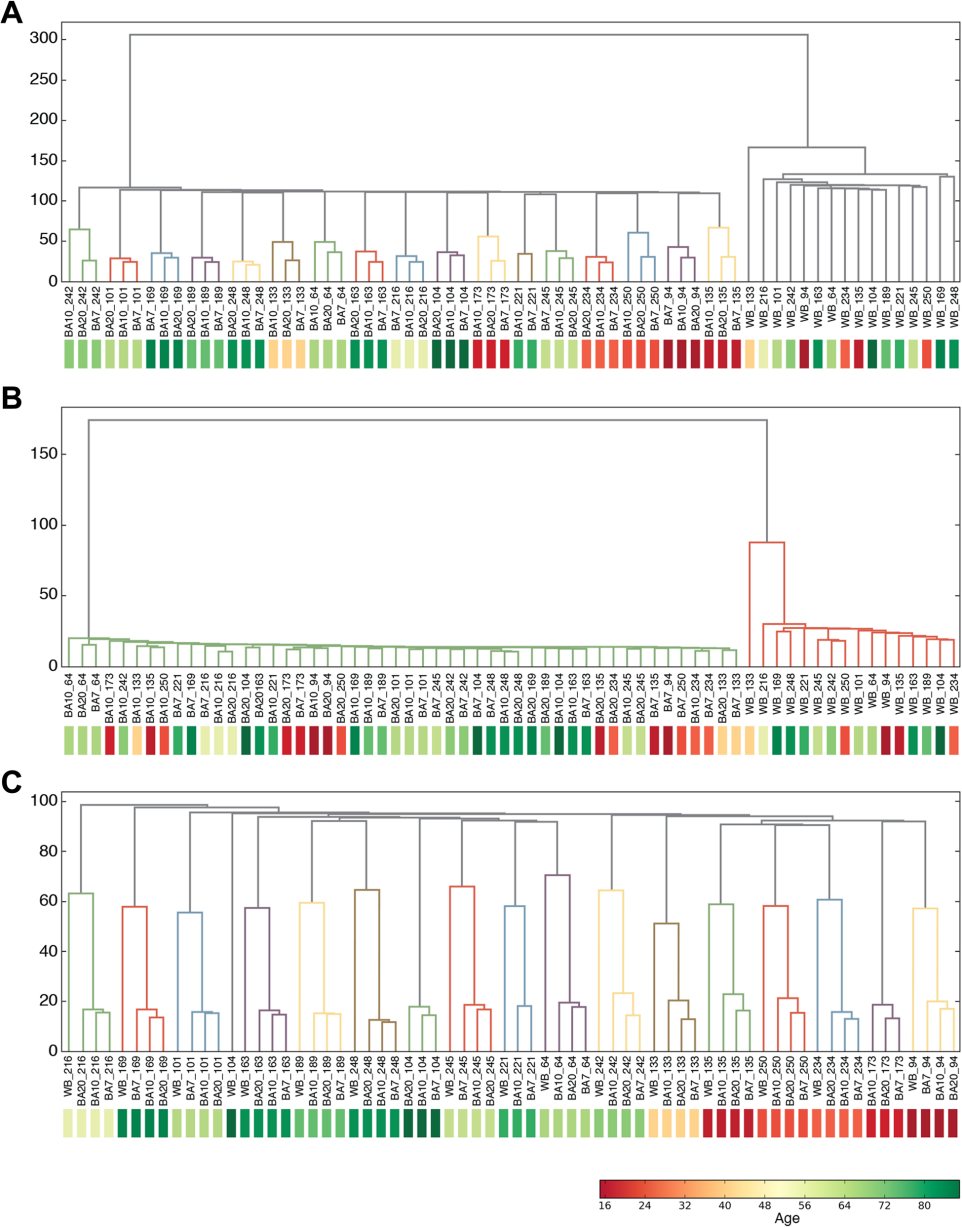
Hierarchical clustering of samples by PCs revealed distinct cluster patterns. Sample IDs indicate sample tissue, color tags (bottom) are mapped to the age of the individual (red: young, green: old). **(A)** Clustering of samples from a selection of all CpG sites with > 4*σ* variance across samples. The first cluster separated blood from brain. Inside the brain region, samples clustered by individual instead of tissue. **(B)** Clustering of samples from a selection of CpG sites with ≷ ± 4*σ* projections in PC1. **(C)** Clustering of samples from a selection of CpG sites with ≷ ± 4*σ* projections in PC4, an age-related PC. PC, principal component; BA10, Broadmann area 10; BA20, Broadmann area 20, BA7, Broadmann area 7; WB, whole blood.

We used hierarchical clustering to further explore how our PCs describe the relationships between samples. To reveal the natural internal relationship between samples, we first computed the nearest neighbor hierarchical clustering similarities of the most highly variable probes in the full dataset. These probes showed variance (>4*σ*) across all samples (7,420 probes) (Figure 3A). The three brain tissues from a given individual clustered together, with BA10 and BA7 being closer to each other and forming a node distinct from BA20. Blood from all individuals clustered separately, and the clustering distance between individuals was generally larger than that in brain (Figure 3A). These data suggested that individual DNA methylation patterns in the three cortical brain regions were more closely related between different individuals than between brain and blood in the same individual, and that inter-individual differences in blood DNA methylation were more pronounced than those in brain DNA methylation. This conclusion is consistent with our analysis of variance presented earlier where blood variance was significantly higher than brain.

We performed clustering using the subset of probes that PCA identified as being correlated with variables of interest to further uncover similarities in DNA methylation between samples. For example, using only the probes that had a projection score ≷ ± 4*σ* on PC1 (2,258 probes), hierarchical clustering separated blood from brain with very few distinctions within the two groups. This approach revealed tissue similarities as the only significant relationship in PC1 regardless of the origin of the individual sample (Figure 3B). This analysis thus confirmed PC1 as a blood vs brain tissue classifier.

A different picture emerged when we performed the hierarchical clustering using only the probes that had a projection score ≷ ± 4*σ* on PC4 (1,993 probes), the PC that showed age-dependent methylation in brain, buccal, and liver, but not blood (Figure 3C). As with the previous examples, this clustering approach confirmed our PCA association as it sorted individuals according to age. In this case, however, brain and blood from the same individual clustered together, with nuanced distinctions within each individual revealing a first order of similarity that encompassed BA10 and BA7, followed by a second node of BA20, and finally a node that included blood. In this case, the probes that contribute to the age-related PC4 showed a stronger effect of individual and a weaker effect of tissue. We previously saw that PC4 was related to age in the brain tissue only, therefore, one may naively expect blood to cluster separately here. However, probes that have strong projections on PC4 can still have significant projections on other individual-specific PCs, as we will show in the following sections. This results in an increased similarity between individuals for this probe subset.

### Tissue and age-dependent methylation profiles were enriched for specific CpG densities

Existing evidence suggests that age-related DNA methylation changes occur at specific genomic locations [14,27,35]. We tested whether the probes that contribute significantly to our top five PCs, all of which were associated with a biological variable, were enriched or depleted for particular genomic regions and CpG island classifications.

**Figure 4:**
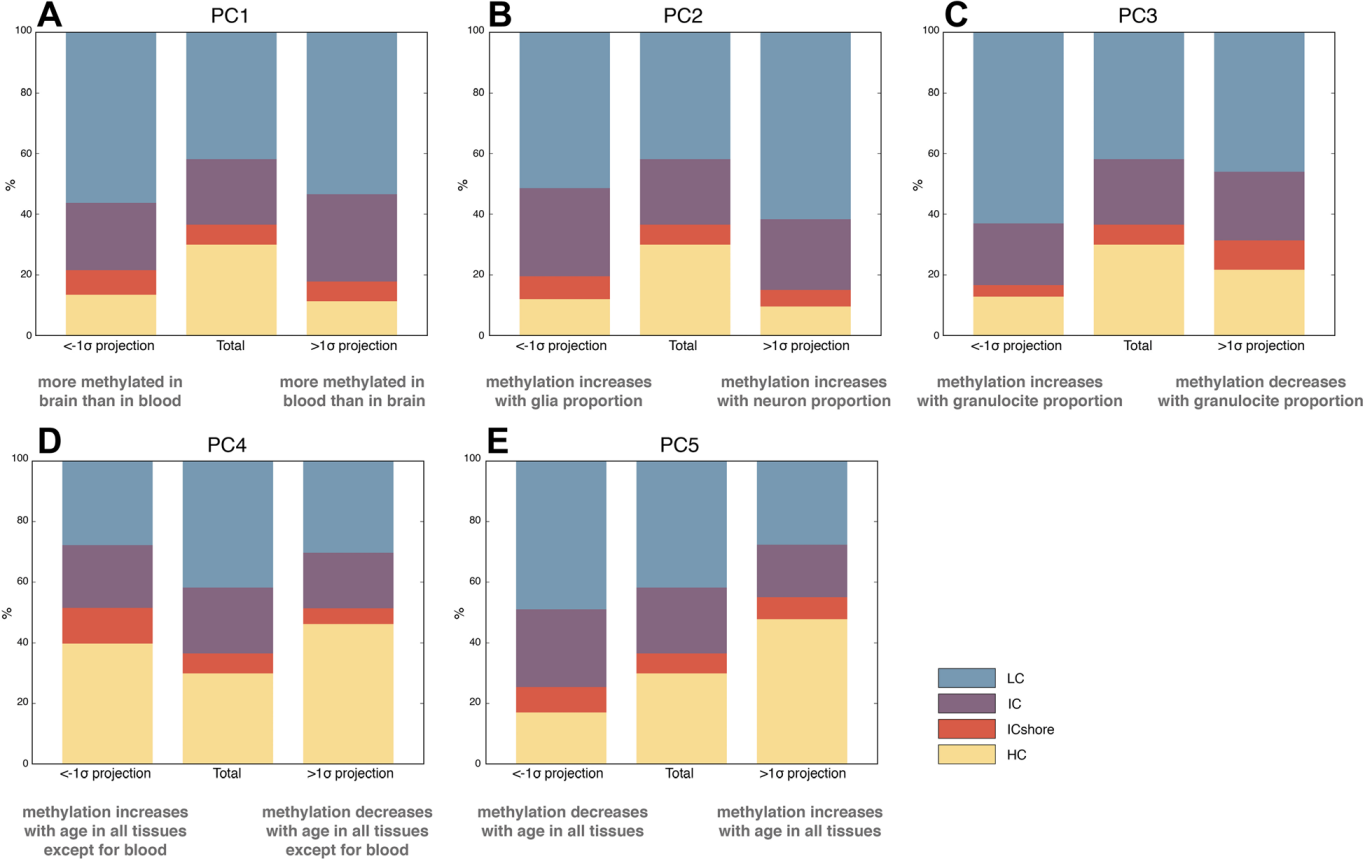
PCs showed distinct CpG density category enrichments. Enrichment and depletion of CpG density categories in subsets of CpG sites with < − 1*σ* projections (left bar) and > 1*σ* projections (right bar) compared to the background total 450K CpG sites (central bar). **(A-C)** Tissue-related PC1 to PC3 showed an enrichment of LC probes and depletion of HC probes irrespective of the sign of the projection. **(D)** PC4, the age pattern present in all tissues checked except blood, showed an enrichment of HC probes and depletion of LC probes in both positive and negative projections. **(E)** PC5, the age pattern present in all the tissues checked, was enriched in HC probes for positive projections (CpGs show increased methylation with age) and was enriched in LC probes for negative projections (CpGs show decreased methylation with age). HC, high-density CpG island; IC, intermediate density CpG island; LC, low-density CpG island; PC, principal component.

CpG loci associated with PCs 1, 2, and 3, which differentiate tissues by its broad origin (blood vs brain for PC1) or cellular heterogeneity within a tissue, (fraction of neuron for PC2; fraction of granulocytes for PC3), respectively, were primarily enriched in low-density CpG (LC) regions and depleted in high-density CpG (HC) contexts (Figure 4A,B,C). This finding was consistent with previous reports that HC islands were less likely to contain tissue-specific, differentially methylated regions [40]. This was true for both positive and negative directions, meaning that the locations of differing DNA methylation did not change depending on whether DNA methylation was higher in one cell or tissue type or the other. A different pattern emerged for CpGs associated with age. CpGs predictive of age in all tissues except blood (PC4) were enriched for HC contexts and depleted in LC contexts, irrespective of whether DNA methylation was gained (negative projections) or lost (positive projections) with age (Figure 4D). While HC enrichment/LC depletion was also found for CpGs associated with age in all tissues (PC5), it was limited to those CpGs where DNA methylation increased with age (Figure 4E). In contrast, CpGs where DNA methylation was lost with age (negative projections) tended to be enriched in intermediate and low CpG contexts and depleted in HC (Figure 4E). This discordance in genomic locations of gain or loss of DNA methylation between the two signatures of age was interesting. The pattern apparent in PC5 showing gain in DNA methylation at HC regions and loss at LC regions was consistent with published reports that HC islands lose DNA methylation with age [14,25,26]. The finding that PC4 had a different pattern, where high-density regions showed both gains and losses in DNA methylation with age, first indicates that the signatures delineated by PCs 4 and 5 were indeed occurring at different sites. Also, since PC4 was not associated with age in blood, it suggested that this pattern of gain of DNA methylation at HC regions may be tissue specific. Together, these results reinforced the idea that these were two independent signatures of age.

We next used a similar approach to address whether subsets of probes are enriched for introns or exons. We observed that probes associated with tissue differentiation (PCs 1 to 3) are enriched for introns and depleted in exons (Additional file 9: Figure S9A,B,C). PC4 showed a slight tendency for the opposite pattern, enrichment for exons and depletion for introns, while PC5 again showed enrichment that was dependent on the sign of the projection score. Positive PC5 scores (increased DNA methylation with age) showed enrichment in exon CpGs, while negative scores (decreased DNA methylation with age) showed enrichment in intron CpGs (Additional file 9: Figure S9D,E).

### CH methylation was associated with specific tissue and cell types

DNA methylation has also been observed at non-CpG sites, sometimes referred to as CH sites [48,49]. Such sites are especially prevalent in the brain. The 450K array contains 3,091 non-CpG probes, thus allowing us the opportunity to determine whether this alternative form of DNA methylation was associated with our variables. In PC1, we observed an enrichment of non-CpG sites for probes with negative projections (probes more methylated in brain than blood, Additional file 10: Figure S10A,F). For PC2, we observed that probes with positive projections were enriched for non-CpG (methylation increases with neuron fraction, Additional file 10: Figure S10B,F). This strongly suggested that non-CpG methylation was not only higher in brain than in blood, but that the brain enrichment was mostly due to neurons as opposed to glia. These two findings were both supported by previous studies, which also show more non-CpG methylation in neurons than other tissues [28]. PC3 showed a general depletion of non-CpG for both projection signs, suggesting that non-CpG sites did not play an important role in white blood cell composition (Additional file 10: Figure S10C,F). The age-related PCs, PC4 and PC5, showed a slight enrichment of non-CpG sites in the probes where methylation increases with age (positive projections in PC4 and negative in PC5, Additional file 10: Figure S10D,F).

### Distinct promoter DNA methylation signatures associated with tissue differentiation and aging

**Figure 5:**
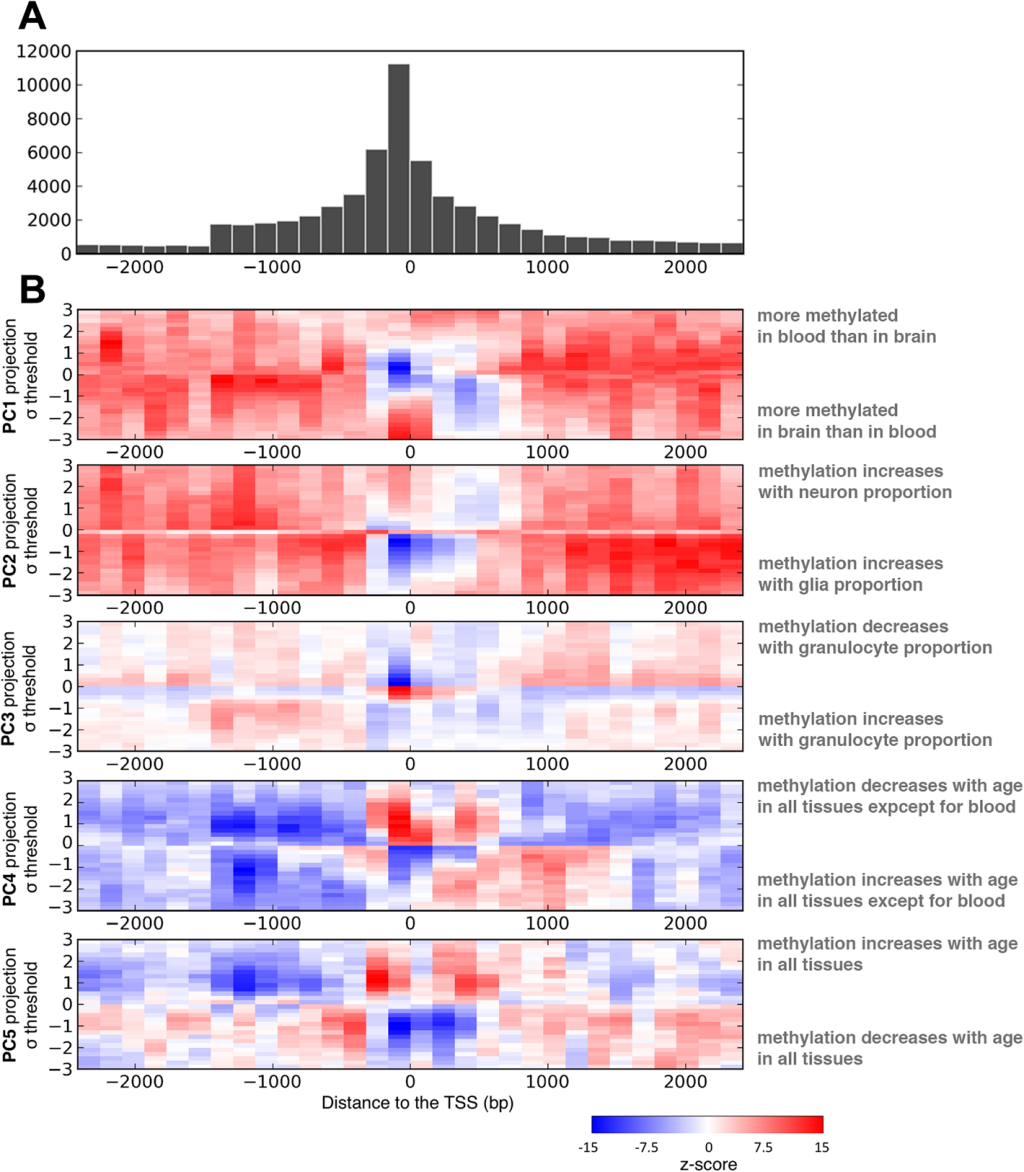
Distinct spatial enrichments of PC-associated CpGs. **(A)** Spatial distribution of CpG probes around the TSS of genes that do not have any neighboring gene in 5Kbp. **(B)** Enrichment Z-scores of probes found at each distance for each PC projection threshold subset of probes, with respect to the background distribution. PC1 to PC3 probes showed a general trend of depletion around the TSS, while they were enriched away from it. PC4 probes were enriched around the TSS and depleted away from it. PC5 probes had a strong sign-dependent trend, positive projections were enriched close to the TSS and negative projections were enriched away from the TSS. PC, principal component; TSS, transcriptional start site.

Given the association between promoter DNA methylation and gene expression, we next explored whether differences in promoter DNA methylation existed between CpGs associated with either tissue differences or age. To unambiguously associate only one promoter with a gene, we focused this analysis on ‘lone genes’ (genes that have no other promoters within 5 kb of the transcriptional start site (TSS), *n* = 16,344). This approach resulted in increased rigor by eliminating sites that might map to more than one gene. We found 60,846 probes on the 450K array that were situated within 2.5 kb distance from the transcription start site (TSS) of a ‘lone gene.’ By design, the content of the 450K array is biased towards CpGs located within 1 kb up and downstream from the TSS (Figure 5A). We used this as a background distribution for evaluating the spatial enrichment of specific probe sets (Additional file 11: Figure S11).

Visualizing the statistically significant locations with respect to the TSS of probes associated with our first five PCs as a heatmap revealed several interesting differences and distinct patterns (Figure 5B). First, tissue- and cell-type-specific CpGs (PC1 to PC3) were generally enriched in regions more than 500 bp away from the TSS. This pattern was especially significant in the brain-related profiles in PC1 and PC2. In PC3, the blood composition pattern, the Z-scores were smaller in magnitude, but still showed this overall trend. We further observed some direction-dependent enrichment. In the neuron-composition PC (PC2), we observed that CpGs where methylation decreases as neuron fraction increases (negative projections) were enriched away from the TSS. For the CpGs where methylation increases with neuron fraction, the Z-scores were not as significant as in the negative projections, but the under-enriched region extended further into the gene itself, and we observed enrichment in proximity to the TSS. These patterns were the same as those observed in PC1 (brain–blood differential methylation). Negative PC1 projections, where the brain tissue was more methylated than blood, showed the same type of enrichment as positive PC2 projections (where neurons are more methylated than glia). This finding suggested that neurons constituted an important source of differential DNA methylation pattern observed in brain vs blood.

We also observed that CpGs predictive of age in all tissues examined except blood (PC4) were generally enriched around the TSS and depleted away from the TSS. These CpGs showed subtle spatial differences depending on a positive or a negative projection. CpGs for which methylation decreases with age in the brain (positive projections) are located close to the TSS (<500 bp away), while probes that increase with age (negative projections) are located further from the TSS (from −1,000 bp to 1,500 bp from the TSS) and are under-enriched at the TSS itself (Figure 5B). Thus, probes located close to the TSS showed a decrease of methylation with age, whereas probes that increase with age tended to be located just upstream of the TSS or within the gene itself.

CpGs predictive of age in all tissues tested (PC5) had a distinct pattern that was dependent on the direction of their component score. CpGs for which DNA methylation increased with age (positive scores) were enriched around the TSS and in proximal regions of the gene body, whereas those for which DNA methylation decreased with age (negative scores) were depleted at the TSS and enriched in upstream and downstream regions (Figure 5B).

**Figure 6:**
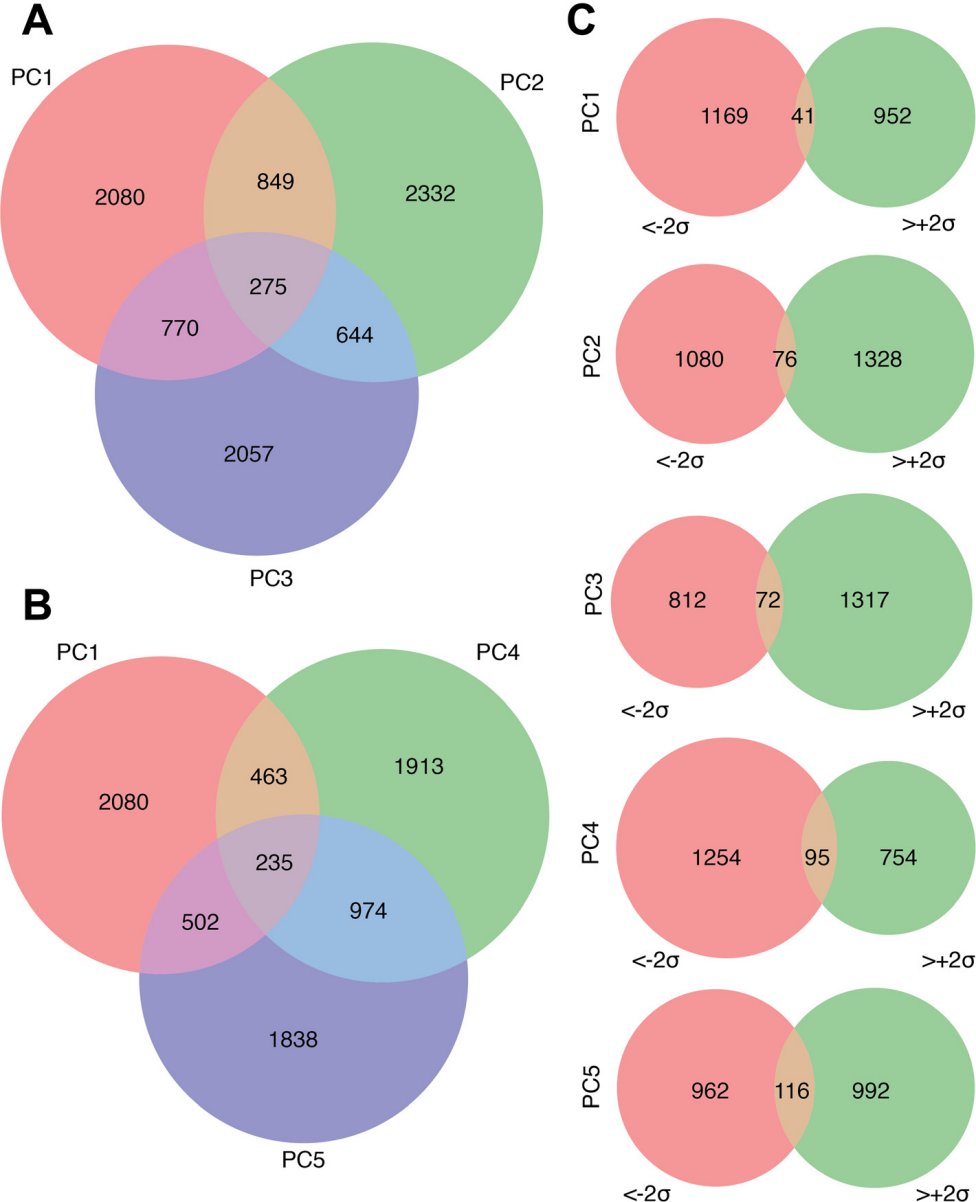
Overlap of genes associated with specific PCs. **(A)** Overlap of genes associated to probes with ≷ ± 2*σ* projection on PCs 1 to 3. **(B)** Overlap of genes associated to probes with ≷ ± 2*σ* projection on PC1 (blood–brain tissue related), PC4 (age in brain), and PC5 (age in brain and blood). **(C)** Overlap of the set of genes that contained probes with > 2*σ* projection with the set of genes with < − 2*σ* projection, for each PC of interest. PC, principal component.

We used our list of 60,846 probes in ‘lone genes’ and determined the overlap in ‘lone genes’ between our various PCs. Using only probes with ≷ ± 2*σ* projection on each PC (an average of 22,221 probes per PC), we determined the overlap of the ‘lone genes’ associated with PCs 1, 2, and 3 and then PCs 1, 4, and 5 (Figure 6A,B). We observed that the overlap between tissue-differentiation genes and aging genes (PC1 ∩ PC4) and (PC1 ∩ PC5) is significantly smaller than the overlap between the two aging sets of genes (PC4 ∩ PC5) (Figure 6B).

### PCs were enriched for biologically relevant gene ontology terms

Lastly, we examined representations of functional categories that were associated with our first five PCs. Before assessing functional enrichments, we examined overlap between positive and negative projections within the same PCs to determine whether they should be examined together or separately. For each PC, we visualized the overlap of the set of genes that contained probes with scores > 2*σ* with the set of genes with score < − 2*σ* (Figure 6C). Taking into account the number of lone genes that contained probes, we calculated the overlap of genes with positive and negative projections that is expected by chance. We found that for the tissue-related PCs (PC1 to 3), the overlap is smaller than expected. This suggests that methylation of tissue-specific probes within the same gene tended to vary in the same direction with tissue or cell type. In contrast, for the age-related PCs, we found an overlap equal to (PC4) and larger (PC5) than expected by chance. Thus, methylation of age-related probes within the same gene can vary in opposite directions with age (Additional file 12: Figure S12).

Gene ontology (GO) analysis using DAVID is presented in Additional file 13: Table S1 [50]. PC1 positive projections referred to genes that were more methylated in blood than in brain and were enriched for clusters including neuron projection and axon morphogenesis and macromolecule catabolism. Negative projections on PC1, which represent genes that were more methylated in brain than in blood, were enriched for clusters that included defense response and response to wounding, and rho signaling. PC2 positive projections had no significantly enriched clusters, while negative projections, representing sites that are more methylated in glia than neurons had three significantly enriched categories, including apoptosis, synaptic transmission, and protein conjugation. For PC3, positive projections denote genes more methylated in granulocytes than non-granulocytes, and were associated with cell motility, inflammation and defense, and secretion and transport. Negative projections, sites that were more methylated in granulocytes than non-granulocytes, were associated with inflammation and defense and metabolism. In all cases, GO terms associated with specific PCs reflect the underlying tissue composition.

For the age-related PCs, PC4 negative projections, reflecting sites that increased with DNA methylation in age in all tissues examined except blood, included a number of categories, including transcription, organ development, neuron cell fate, adhesion, and differentiation. PC4 positive projections had no significant associations. For PC5 positive projections, representing sites for which DNA methylation increased with age in all tissues, associated clusters included developmental processes and transcriptional regulation. PC5 negative projections had no enrichment. Thus, both age-related PCs had no functional enrichment categories for sites that lost DNA methylation with age, while both contained categories related to development and differentiation in sites that gain DNA methylation with age. This was consistent with previously published reports [25,29].

## Conclusions

Using matched tissues from different individuals, we have shown that PCA is capable of simultaneously identifying independent DNA methylation signatures of a number of variables of interest. This approach is particularly helpful for those variables which are related to one another, such as age and white blood cell composition [16]. By testing the correlation of each variable with each PC, it is possible to find PCs where one variable is correlated, but the other is not. We were able to find PCs associated with age that were not correlated with white blood cell composition, which is known to be a major difficulty in assessing age-related DNA methylation from blood [16]. These results have implications for other studies by indicating the relative contributions of factors known to cause changes in DNA methylation pattern. Our results indicated that tissue, cell type, and age, in that order, all had very important effects on DNA methylation. Future development of this method could target clusters of CpGs rather than individual CpGs, identifying broader regions of variable-associated differential methylation.

Principal component analysis has been extensively used in gene expression studies. We show here that PCA can provide functionally relevant insight into DNA methylation variation as well. The use of a PCA-based approach allowed us to overcome difficulties often associated with epigenetic studies. The most common method for analyzing DNA methylation data is linear modeling. PCA complements from linear modeling, but differs in two primary ways: first, PCA does not assume a particular linear (or non-linear) relationship between the variable of interest and the DNA methylation profile and second, PCA uses a Z-score to assign a confidence to how strongly a particular PC accounts for a given CpG’s methylation pattern. These differences in many cases can result in benefits to performing PCA. For example, one potential caveat with our study was small sample size, which might not be conducive to deriving generally applicable relationships, particularly using traditional methods such as linear modeling. However, the reproducibility of PCs derived from only 17 subjects in a much larger cohort suggests that our PCA approach is an excellent tool to detect a meaningful association between DNA methylation and biological variables even when only small sample sizes are available. This work then highlights the issue of statistical power calculations in epigenetic research, which has practical relevance for the design of epigenetic studies. It should also be noted that both the white blood cell preparation and the brain samples used here constitute heterogeneous mixtures of cell types. Since we were able to use established methods to predict the cellular composition of both tissues, we could identify specific PCs that associated with cellular composition and reassure ourselves that our aging signatures in brain and brain–blood did not correlate with differences in cellular composition. Finally, it is important to note that PCs 1 and 3 might have a contribution of hydroxymethylation to the signal. We cannot unambiguously determine whether hydroxymethylation contributes positively or negatively to these PCs, and ultimately how it relates to CpG density, promoter spatial enrichment, or pathway analysis. As hydroxymethylation is generally not found at promoter regions; it likely has a smaller effect on our determinations of promoter spatial enrichment [4,51].

Our analysis provides a map of broad patterns of DNA methylation in two important tissues, and laid some important ground work on how these patterns were similar and different across tissues. Future work will be required to ferret out whether these broad patterns have functional implications. Interestingly, our data provided evidence for the existence of at least two independent DNA methylation signatures associated with age. The first signature was observed in all tissues examined, while the second was found in all except blood. More broadly, this suggested that epigenetic signatures even for the same variable could be both tissue specific and tissue independent. This is particularly relevant as blood and brain originate from different germ layers. Future research in larger cohorts with carefully ascertained cognitive variables might reveal potential linkages between these types of DNA methylation signatures and cognition.

## Methods

### Data collection

DNA was extracted from samples from the Quebec Suicide Brain Bank using the Qiagen DNAeasy DNA extraction kit (Qiagen, Valencia, CA, USA). Brain tissue was obtained from the Douglas-Bell Canada Brain Bank (DBCBB; Douglas Mental Health University Institute, Montréal, Québec). All subjects were psychiatrically diagnosed by means of psychological autopsy, which is a validated method to reconstruct psychiatric history by means of extensive proxy-based interviews [52]. Individuals were Caucasian and died suddenly, with no prolonged agonal period. Exclusion criteria were a lifetime trauma exposure, a current DSM-IV axis I psychiatric diagnosis including any form of substance abuse [53]. Brain tissue dissection was carried out as previously described [39]. Briefly, tissues from the left hemisphere were carefully dissected at 4°C after having been flash-frozen in isopentene at −80°C. Brain tissue was dissected and Brodmann areas (BA) identified using reference neuroanatomical maps. The Research Ethics Board at the Douglas Mental Health University Institute approved the project. Signed informed consent was obtained for each subject from next of kin.

DNA was treated with sodium bisulfite using the Zymo EZ-DNA kit (Zymo Research, Orange, CA, USA) according to manufacturer’s instructions. All samples were randomized before bisulfite treatment, then randomized again before beginning the Illumina array protocol. DNA was processed and hybridized to a total of six of the Illumina Infinium HumanMethylation 450 BeadChips (Illumina Inc., CA, USA) according to manufacturer’s instructions, then scanned on an Illumina HiScan (Illumina Inc., CA, USA). After scanning, data was imported into Genome Studio and control probes were examined to ensure data quality, after which data was exported into R. Next, probes were filtered to remove any probes on the X and Y chromosomes (11,648), probes for which any sample showed a detection *P* value greater that 0.01 or fewer than three beads contributing to the signal (27,541), the 65 SNP genotyping probes, and probes that assess polymorphic CpGs or that cross-hybridize to the X or Y chromosomes, leaving a total of 408,576 probes remaining [3]. Background subtraction, color correction, and quantile normalization were performed on all samples together using the lumi R package, and peak-based correction was used to normalize Type I and Type II probes [54,55]. At this point, M values were exported from R for further analysis using Python.

### Principal component analysis

PCA is a mathematical approach that reveals the internal structure of variation in a data matrix. It calculates a set of principal components of variation, along with a set of associated eigenvalues that quantify how much variation is captured by each principal component (Figure 1A and Additional file 1: Figure S1A).

We built an N×M matrix of M values, X, where each row is a CpG from Illumina 450K Human DNA methylation array, and each column is a collected sample. The mean M-value of each column, $\overline{x},$ was subtracted (Additional file 1: Figure S1B). Next, the M×M covariance matrix was calculated from the data and was diagonalized, getting the corresponding matrix of eigenvectors V (principal components, PCs) and eigenvalues ${\sigma}_i^2$ (variance associated to each PC). Each PC is an M-long vector.

M values are distributed bimodally over the entire collection of CpG probes. This highlights that there are low methylated probes and high ones. Since PCA calculates the dominant contributions to the variance, this high-low variation forms the zeroth PC. It is merely a constant offset that shifts the mean methylation from one probe to the next (Additional file 1: Figure S1C) and accounts for 96% of the variation. We subtracted out this contribution and considered only the variation in methylation across samples after this constant offset was taken account of.

To assess potential batch effects, we performed a Kolmogorov-Smirnov test to determine whether bead chip or position on the bead chip affected distributions of PC scores. After Bonferroni correction at a *P* value cutoff of 0.01, no chip or position on the chip showed significantly different scores for PCs 1 to 5, indicating little contribution of batch in our variable-associated PCs (Additional file 14: Figure S14).

### Projection thresholding

The projection of each CpG site onto the eigenvector matrix (*P* = *XV*) quantifies how much each PC contributes to the pattern seen on the CpG site. Projection values are approximately normally distributed with zero mean and variance equal to the associated eigenvalue. The CpG sites with the largest positive projections on a given PC represent the CpG sites where the patterns resemble the PC profile. In contrast, large negative projections correspond to the patterns described by the inverted PC (that is, each component of the PC multiplied by −1) (Additional file 3: Figure S3).

The selection of the subset of CpGs with the greatest projections allows us to study the CpG sites responsible for driving the pattern of interest. We consider a probe to have a ± *nσ* contribution from a given PC if its projection is ≷ ± *nσ*_*i*_, where ${\sigma}_i^2$ is the eigenvalue of the associated PC (Additional file 3: Figure S3).

### Principal component reconstruction

Ideally, we seek to associate the patterns of methylation for each PC with an observable trait such as age, tissue, and so on. We can consider the projections as representing the strength of a ‘vote’ of a given CpG for that particular PC. If a PC is associated with a trait, we can construct a trait predictor using these projections. Given the data matrix *X* and the projection matrix *P* we can reconstruct the matrix of PCs *V* using *V* = norm(*X*^**T**^*P*) where norm indicates normalization of columns to unity.

The value of this approach is to reconstruct the associated trait predictors (PCs) in an unknown dataset *Y*, by using the associated votes from each CpG from the known data *X*. By analogy to the expression above, the new reconstructed matrix of PCs *Ṽ* is defined as *Ṽ* = norm(*Ỹ*^**T**^*P*).

### Blood and brain composition

The neuron/glia composition of our brain samples was computed using the publicly available CETS package for R [42]. To obtain the cell composition of our blood samples we used a published deconvolution method [18,41].

### Hierarchical clustering

To perform hierarchical clustering, we computed the sample similarities using the nearest point algorithm: $$ \mathrm{d}\left(u,v\right)= \min \left(\mathrm{d}\left({\overrightarrow{u}}_i,{\overrightarrow{v}}_j\right)\right) $$, where d is the Euclidian distance, min is the minimum, *u* and *v* are two different clusters, and $$ {{\overrightarrow{u}}_i}_{,} $$$$ {\overrightarrow{v}}_j $$ are vectors from the respective clusters. The dendogram was calculated and plotted using the ‘scipy.cluster.hierarchy’ Matplot library from Python.

### Enrichment of spatial location around the TSS

From the hg19 human genome release, we selected genes that do not have any neighboring TSS within 5 kbp distance from their own TSS. We found 16,344 genes meeting this criterion, which we call ‘lone genes.’ In order to discard methylation interference from neighboring genes, we selected probes whose distance to the lone genes’ TSS is smaller than 2.5 kbp. The number of Illumina 450K Human DNA methylation probes associated to lone genes was 60,846 after correcting for poorly performing probes, probes with SNPs, probes hybridized to multiple locations in the genome, and those located on the X and Y chromosomes. These probes mapped to 10,176 out of the total 16,344 ‘lone genes.’

For a given a subset of probes, we compared their spatial distribution with that of the background distribution of probes associated with the lone genes (*N*_bg_ = 60, 486). We selected the subset of probes that have a certain threshold projection onto the PC of interest, with size *N*_exp_. The distances of each probe from the TSS were binned (bin size = 167 bp). For each bin, this gave the number of probes from the enriched set *n*_exp_ and background *n*_bg_ To obtain an enrichment Z-score, we considered the null distribution of the enrichment set to be a binomial with mean *μ* and variance *σ*^2^ with probability *p* = *n*_bg_/*N*_bg_:

$$ \mu =\frac{n_{\mathrm{bg}}}{N_{\mathrm{bg}}}{N}_{\exp } $$

$$ {\sigma}^2=\left(1-\frac{\mu }{N_{\exp }}\right)\mu $$

$$ Z=\frac{\left({n}_{\exp }-\mu \right)}{\sigma } $$

### Gene ontology analysis

All the CpG sites that are less than 2.5 kbp away from the TSS of a lone gene were associated to the corresponding gene. We have calculated enrichments for biological functions by comparing the features of the genes contained in a particular CpG subset, to the background (‘lone genes’ that have probes on them). The GO terms were obtained using DAVID 6.7 [50,56].

## Additional files

Additional file 1: Figure S1. Principal component summary. **(A)** Cumulative sum of variance % of each PC. We can observe that 75% of the total variance is captured by PC1. Ninety percent of the total variation is captured by the first 13 PCs. **(B)** Mean methylation M-value of each sample. **(C)** PC0 emerged as the most dominant pattern. It had a horizontal line shape since it is due to a methylation offset between CpGs that have high values across all samples and CpGs that have low values. **(D)** PC6 showed variability across individuals but not across tissues and it was not correlated with any of the variables measured. **(E)** PC27 showed tissue-related levels of methylation for BA7 samples. BA10, Broadmann area 10; BA20, Broadmann area 20, BA7, Broadmann area 7; PC, principal component; WB, whole blood. Additional file 2: Figure S2. Scatter plot of PCs vs sample features. PC1 was correlated with tissue of origin. PC2 was correlated with neuron proportion in brain samples. PC3 was correlated with granulocyte proportion of whole blood samples. PC4 was correlated with age in brain samples but not in whole blood samples. PC5 was correlated with age in both brain and whole blood samples. BA10, Broadmann area 10; BA20, Broadmann area 20, BA7, Broadmann area 7; PC, principal component; WB, whole blood. Additional file 3: Figure S3. Projection thresholding. (Top) Histogram of the 450K probe projections for PC1. (Bottom) Six probes with different positive and negative scores. Positive scoring probes are more methylated in the blood than in the brain, whereas negative scoring probes are more methylated in the brain than in the blood. The similarity between the probe profile and PC1 (Figure 1A) increases with the magnitude of the score value. PC, principal component. Additional file 4: Figure S4. Variance across samples in each tissue. Line plots show the variance distributions; bar plots show the percentage of total variance on each tissue. **(A)** Original data. **(B)** Data without cell type contribution. Blood was significantly more variable across samples than brain. BA10, Broadmann area 10; BA20, Broadmann area 20, BA7, Broadmann area 7; WB, whole blood. Additional file 5: Figure S5. PCs for which blood patterns and brain patterns correlated with each other. This subset of PCs captured 33.1% of the non-tissue-specific variation (PCs after PC3). The first 8 patterns have positive correlations and they capture 74.5% of the variance of the correlating subset; the following 11 patterns have negative correlations and represent 25.5% of the variance of the subset. PC, principal component. Additional file 6: Figure S6. Correlation of blood and brain methylation patterns. We show the variance captured by individual-specific PCs (PCs after PC3) classified by the correlation of DNA methylation patterns in the brain and blood. Additional file 7: Figure S7. PCA performed on each tissue separately. All brain tissues (BA10, BA20, BA7) showed a first PC that correlated with neuron proportion **(A-C)** and a second one that correlated with age **(E-G)**. Whole blood (WB) samples showed two PCs that correlated with granulocyte proportion **(D, H)** and no PCs correlating with age of participants (not shown). BA10, Broadmann area 10; BA20, Broadmann area 20, BA7, Broadmann area 7; PC, principal component; WB, whole blood. Additional file 8: Figure S8. Percentage of datasets with PCs with a significant correlation (*P* < 0.05 and *P* < 0.01) with the age of participants after cell composition was subtracted. The datasets of different sizes were generated with random sampling from a larger cohort. We observed that an age PC can be found with a probability of approximately 60% only in datasets with >22 samples. Additional file 9: Figure S9. Enrichment and depletion of intron/exon categories in subsets of CpG sites with < − *σ* projections (left bar) and > *σ* projections (right bar) compared to the background total 450K CpG sites (central bar). **(A-C)** Tissue-related PC1-PC3 showed an enrichment of intron probes and depletion of exon probes irrespective of the sign of the projection. **(D)** PC4, the age signature found in all tissues except blood showed an enrichment of exon probes and depletion of intron probes in both positive and negative projections. **(E)** PC5, the age signature that was found present in all of the tissues checked was found enriched in exon probes in positive projections (CpGs increase methylation with age) and enriched in intron probes in negative projections (CpGs decrease methylation with age). PC, principal component. Additional file 10: Figure S10. Enrichment and depletion of CpG/non-CpG categories in subsets of probes with < − *σ* projections (left bar) and > *σ* projections (right bar) compared to the background total 450K probes (central bar). PC1 showed an enrichment of non-CpG sites for probes with negative projections (probes more methylated in brain than blood) **(A)**. For PC2, probes with positive projections were enriched for non-CpG (methylation increases with neuron fraction) **(B)**. PC3 showed a general depletion of non-CpG for both projection signs **(C)**. The age-related PCs, PC4 and PC5, showed a slight enrichment of non-CpG sites in the probes where methylation increases with age (positive projections in PC4 and negative in PC5) **(D,F). (E)** Projections of non-CpG sites in each PC normalized by the standard deviation of all 450K probe projections in the PC. PC, principal component. Additional file 11: Figure S11. Scheme of the construction of spatial enrichment heatmaps. In gray we show the background number of probes in each distance bin. Error bars show the standard deviation of a binomial distribution. The color plot is a hypothetical example of an experimental distribution of probes. The number of standard deviations away from the mean background (Z-score) is mapped to a color, where red corresponds to enrichment and blue corresponds to depletion. TSS, transcriptional start site. Additional file 12: Figure S12. Overlaps of genes containing probes with positive and negative 2*σ* projections on the first five PCs. The independent-case overlaps were calculated as the product of the observed probabilities of belonging to each gene set. Additional file 13: Table S1. List of GO term enrichment results from DAVID. Additional file 14: Figure S14. Distribution of PC scores sorted by chip and row position for the evaluation of possible batch effects. Kolmogorov-Smirnov tests indicated that all distributions were significantly similar. BA10, Broadmann area 10; BA20, Broadmann area 20, BA7, Broadmann area 7; PC, principal component; WB, whole blood.

## Abbreviations

BA7: Broadmann area 7
BA10: Broadmann area 10
BA20: Broadmann area 20
GO: Gene ontology
HC: High-density CpG island
IC: Intermediate density CpG island
KS: Kolmogorov-Smirnov
LC: Low-density CpG island
PCA: Principal component analysis
PC: Principal component
TSS: Transcriptional start site
