# Supplementary figures and images for: Concordant and discordant DNA methylation signatures of aging in human blood and brain
Source: Epigenetics Chromatin. 2015 May 9;8:19. doi: 10.1186/s13072-015-0011-y (PMC4430927; doi:10.1186/s13072-015-0011-y)

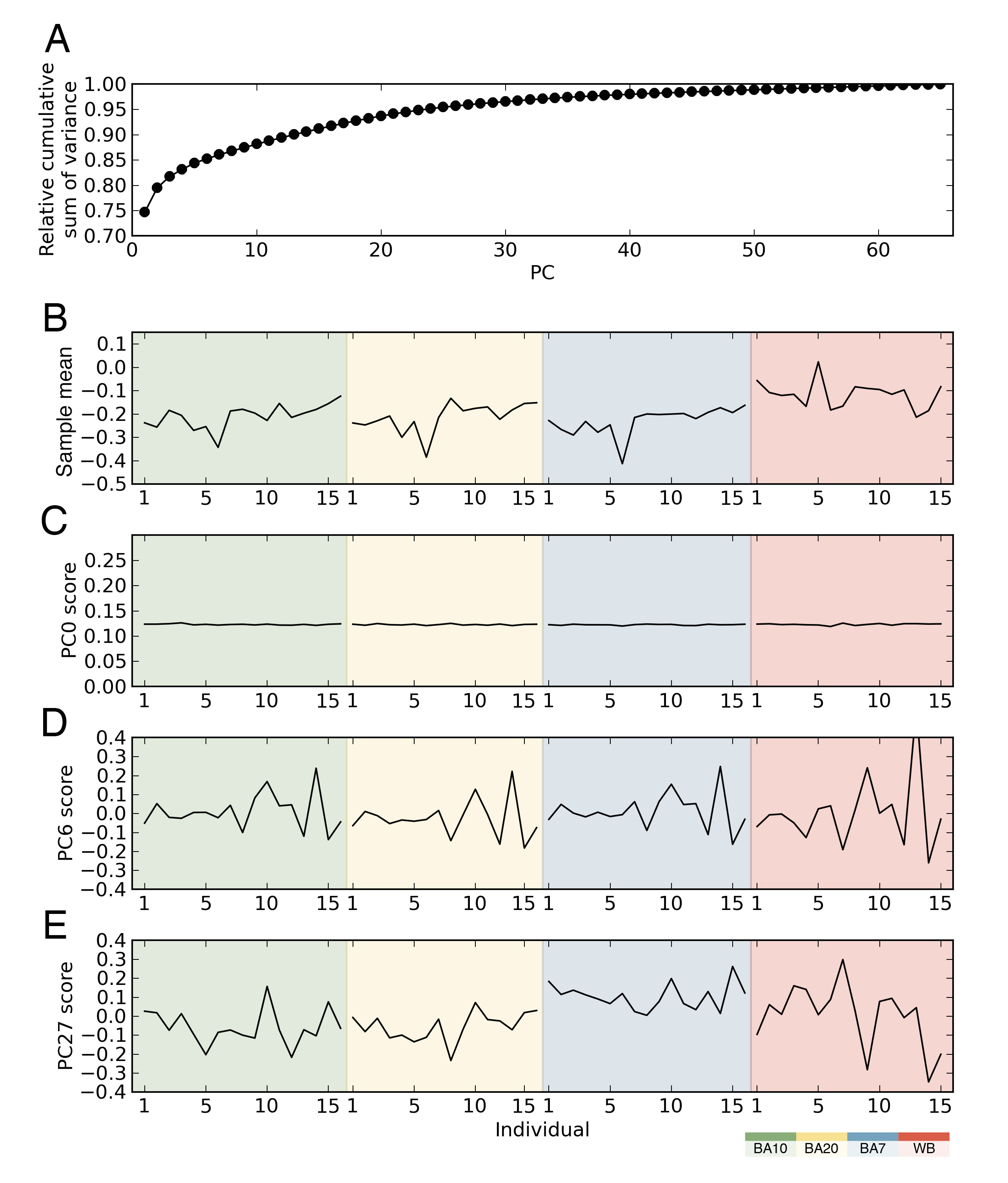

Supplement: Additional file 1: Figure S1. — Principal component summary. (A) Cumulative sum of variance % of each PC. We can observe that 75% of the total variance is captured by PC1. Ninety percent of the total variation is captured by the first 13 PCs. (B) Mean methylation M-value of each sample. (C) PC0 emerged as the most dominant pattern. It had a horizontal line shape since it is due to a methylation offset between CpGs that have high values across all samples and CpGs that have low values. (D) PC6 showed variability across individuals but not across tissues and it was not correlated with any of the variables measured. (E) PC27 showed tissue-related levels of methylation for BA7 samples. BA10, Broadmann area 10; BA20, Broadmann area 20, BA7, Broadmann area 7; PC, principal component; WB, whole blood. [file 13072_2015_11_MOESM1_ESM.png]

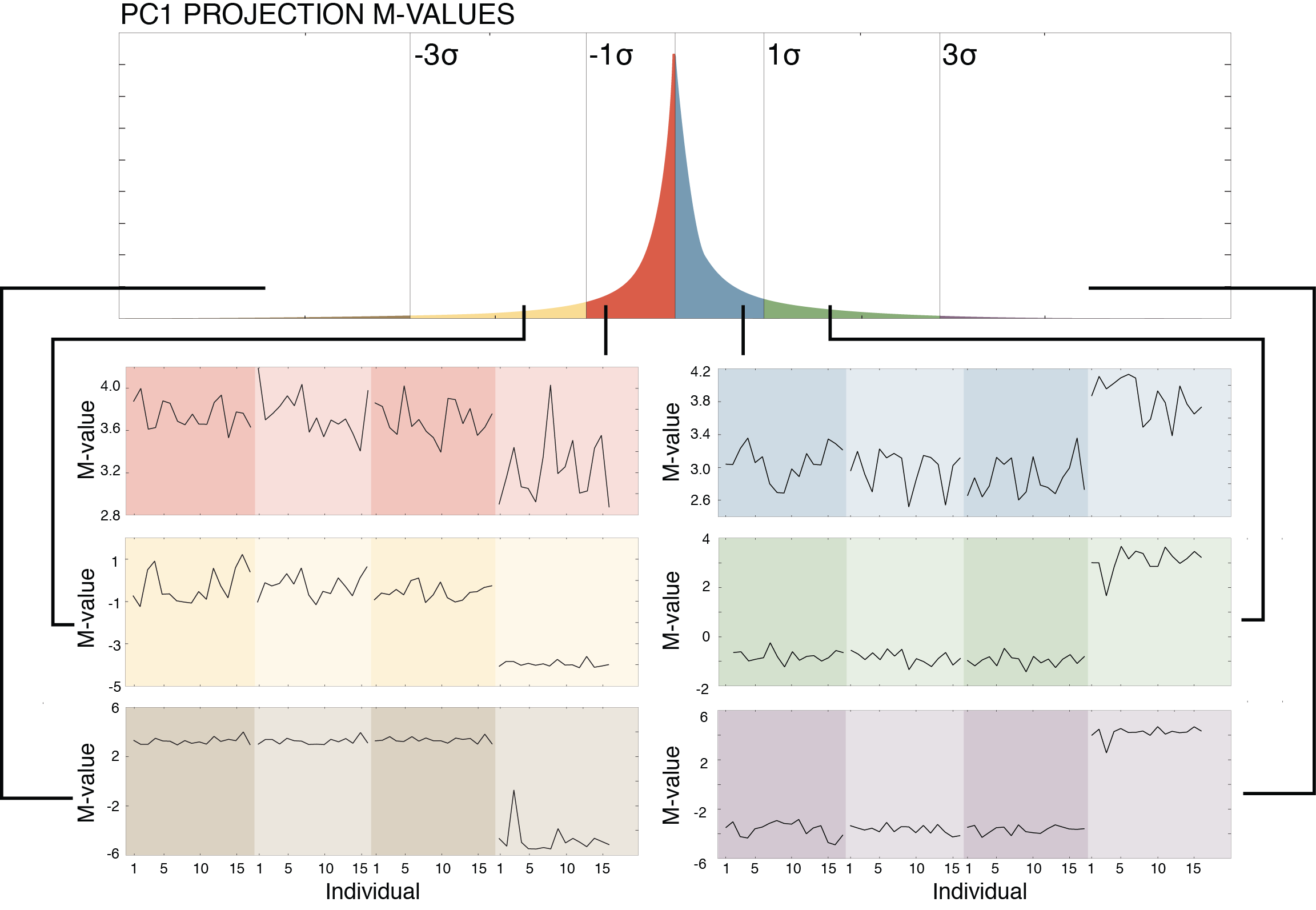

Supplement: Additional file 3: Figure S3. — Projection thresholding. (Top) Histogram of the 450K probe projections for PC1. (Bottom) Six probes with different positive and negative scores. Positive scoring probes are more methylated in the blood than in the brain, whereas negative scoring probes are more methylated in the brain than in the blood. The similarity between the probe profile and PC1 (Figure 1A) increases with the magnitude of the score value. PC, principal component. [file 13072_2015_11_MOESM3_ESM.png]

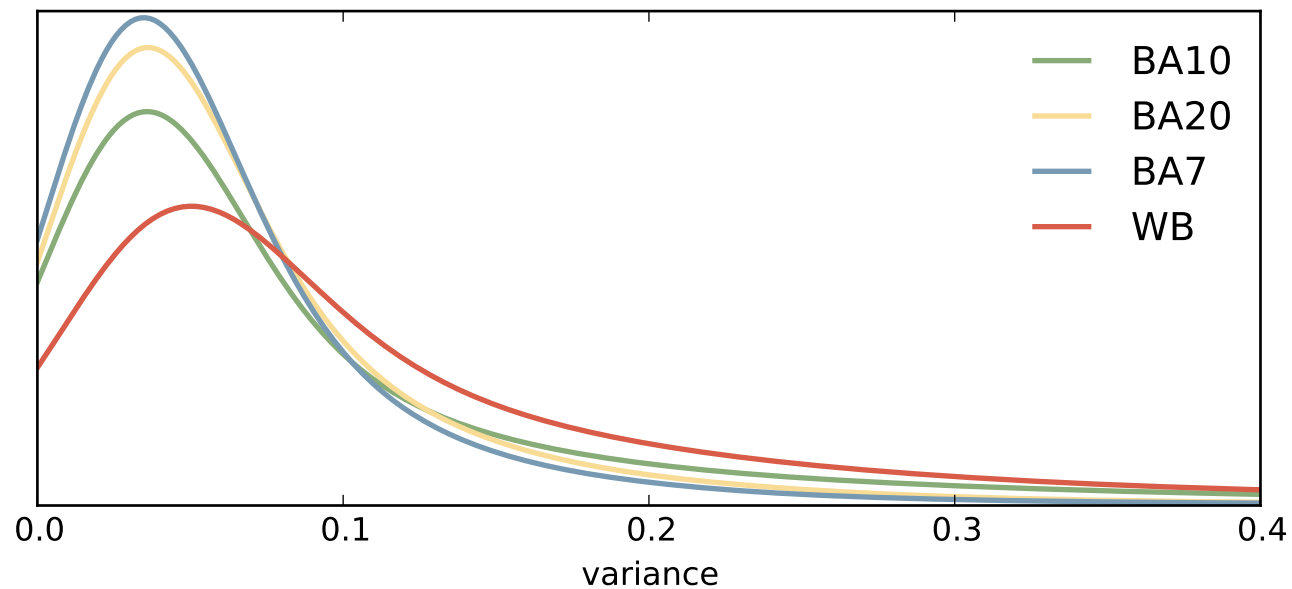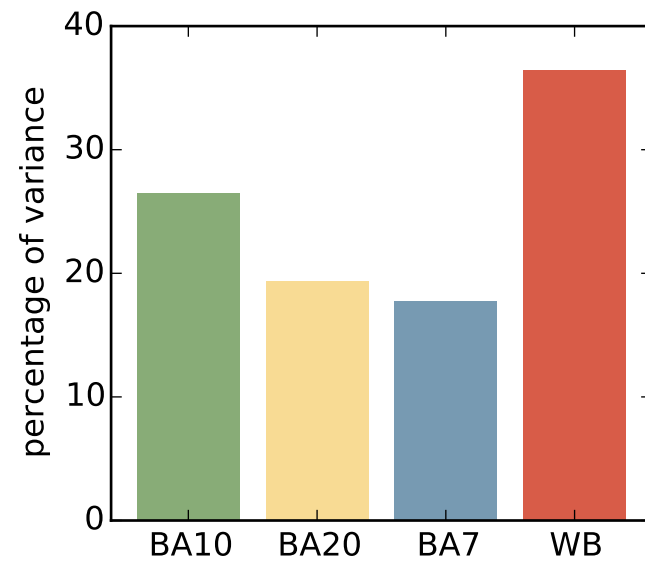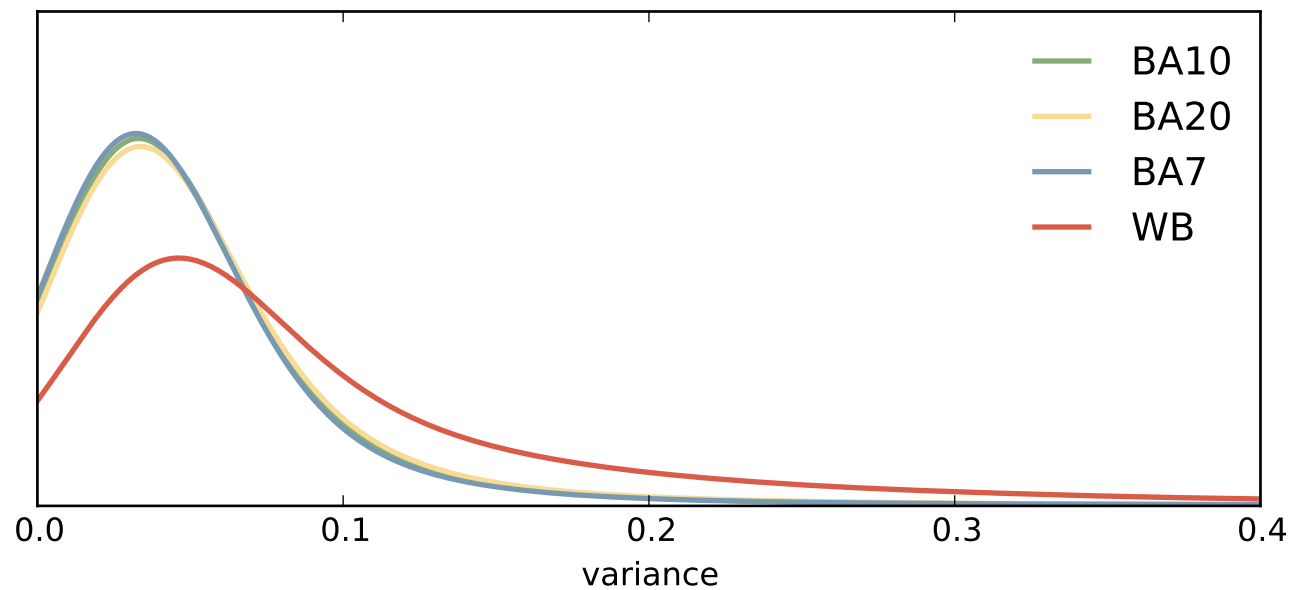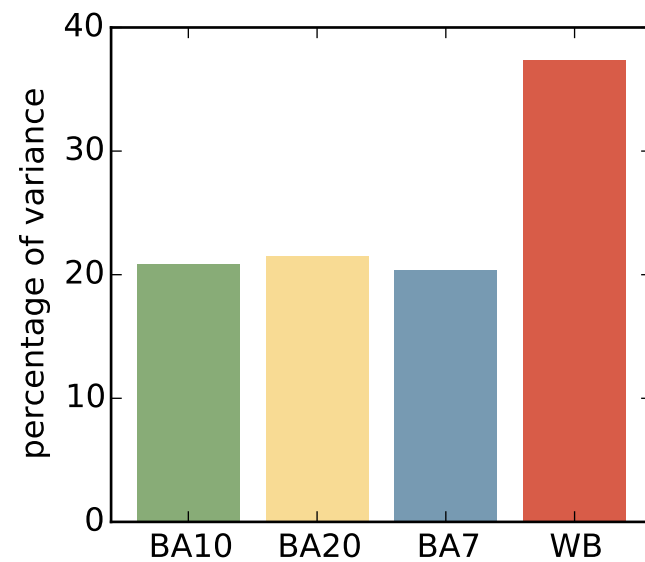

Supplement: Additional file 4: Figure S4. — Variance across samples in each tissue. Line plots show the variance distributions; bar plots show the percentage of total variance on each tissue. (A) Original data. (B) Data without cell type contribution. Blood was significantly more variable across samples than brain. BA10, Broadmann area 10; BA20, Broadmann area 20, BA7, Broadmann area 7; WB, whole blood. [file 13072_2015_11_MOESM4_ESM.pdf]

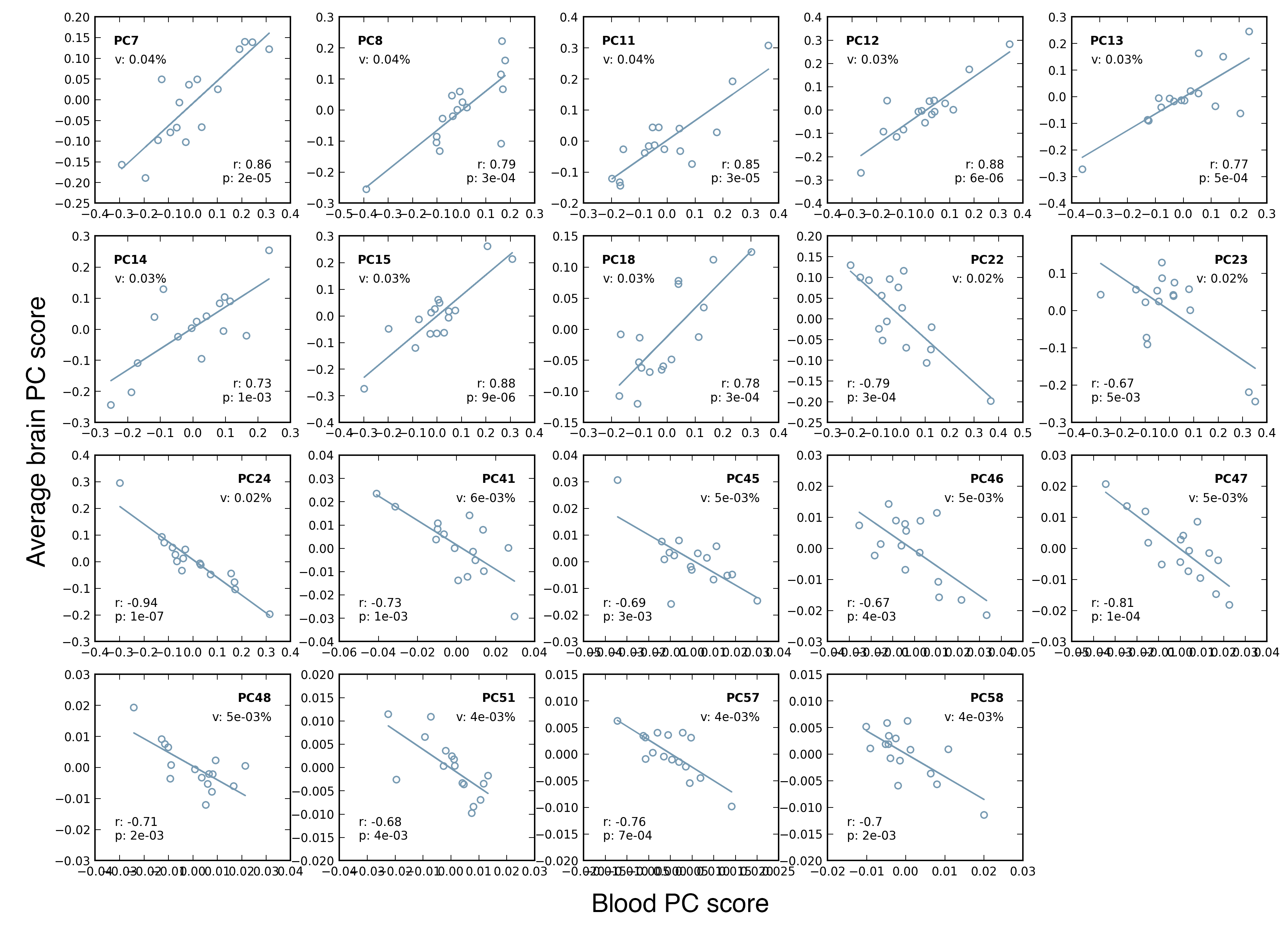

Supplement: Additional file 5: Figure S5. — PCs for which blood patterns and brain patterns correlated with each other. This subset of PCs captured 33.1% of the non-tissue-specific variation (PCs after PC3). The first 8 patterns have positive correlations and they capture 74.5% of the variance of the correlating subset; the following 11 patterns have negative correlations and represent 25.5% of the variance of the subset. PC, principal component. [file 13072_2015_11_MOESM5_ESM.png]

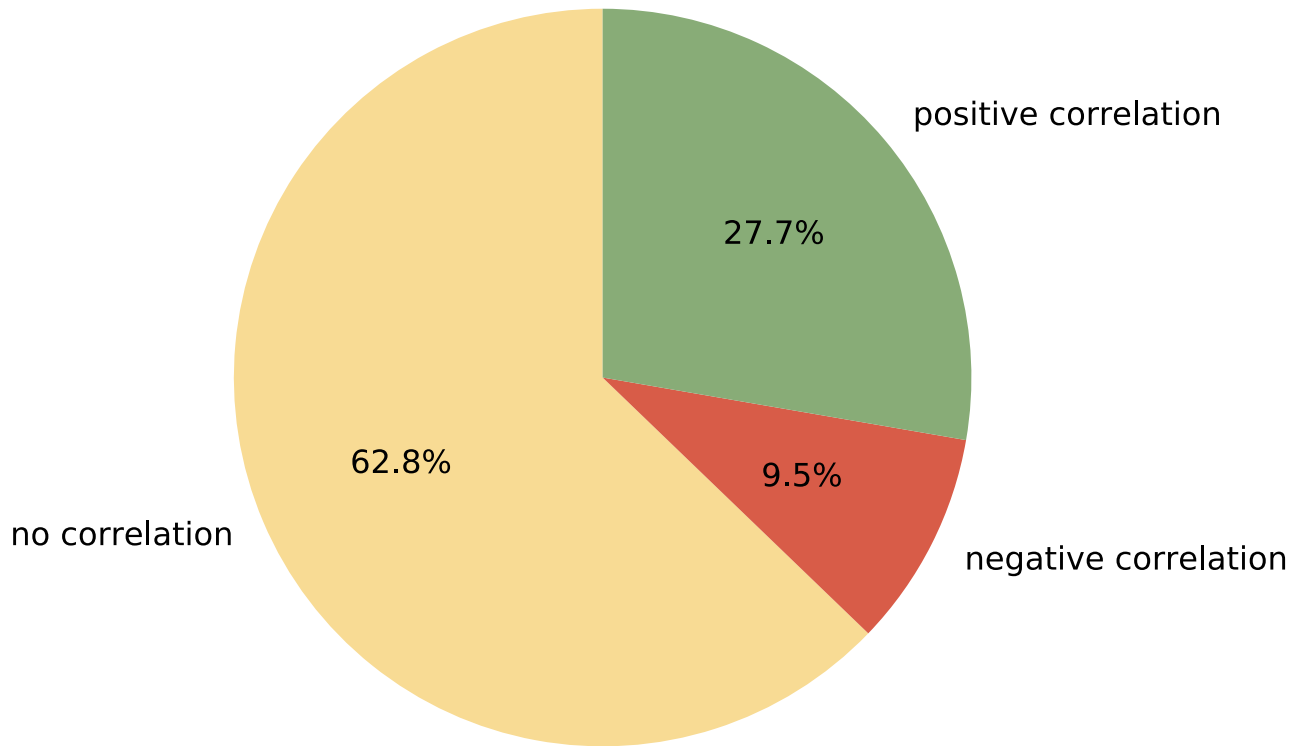

Supplement: Additional file 6: Figure S6. — Correlation of blood and brain methylation patterns. We show the variance captured by individual-specific PCs (PCs after PC3) classified by the correlation of DNA methylation patterns in the brain and blood. [file 13072_2015_11_MOESM6_ESM.pdf]

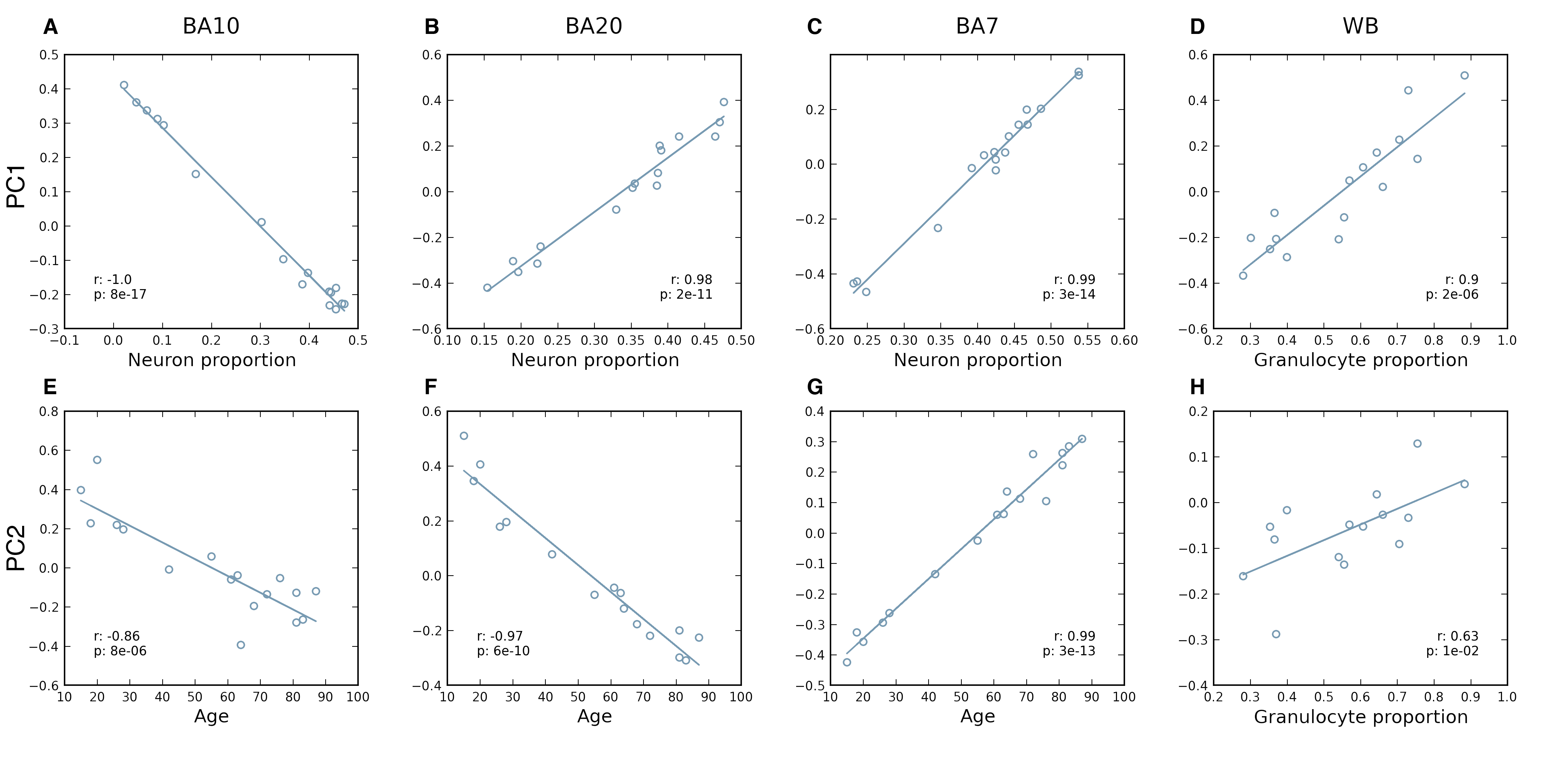

Supplement: Additional file 7: Figure S7. — PCA performed on each tissue separately. All brain tissues (BA10, BA20, BA7) showed a first PC that correlated with neuron proportion (A-C) and a second one that correlated with age (E-G). Whole blood (WB) samples showed two PCs that correlated with granulocyte proportion (D, H) and no PCs correlating with age of participants (not shown). BA10, Broadmann area 10; BA20, Broadmann area 20, BA7, Broadmann area 7; PC, principal component; WB, whole blood. [file 13072_2015_11_MOESM7_ESM.png]

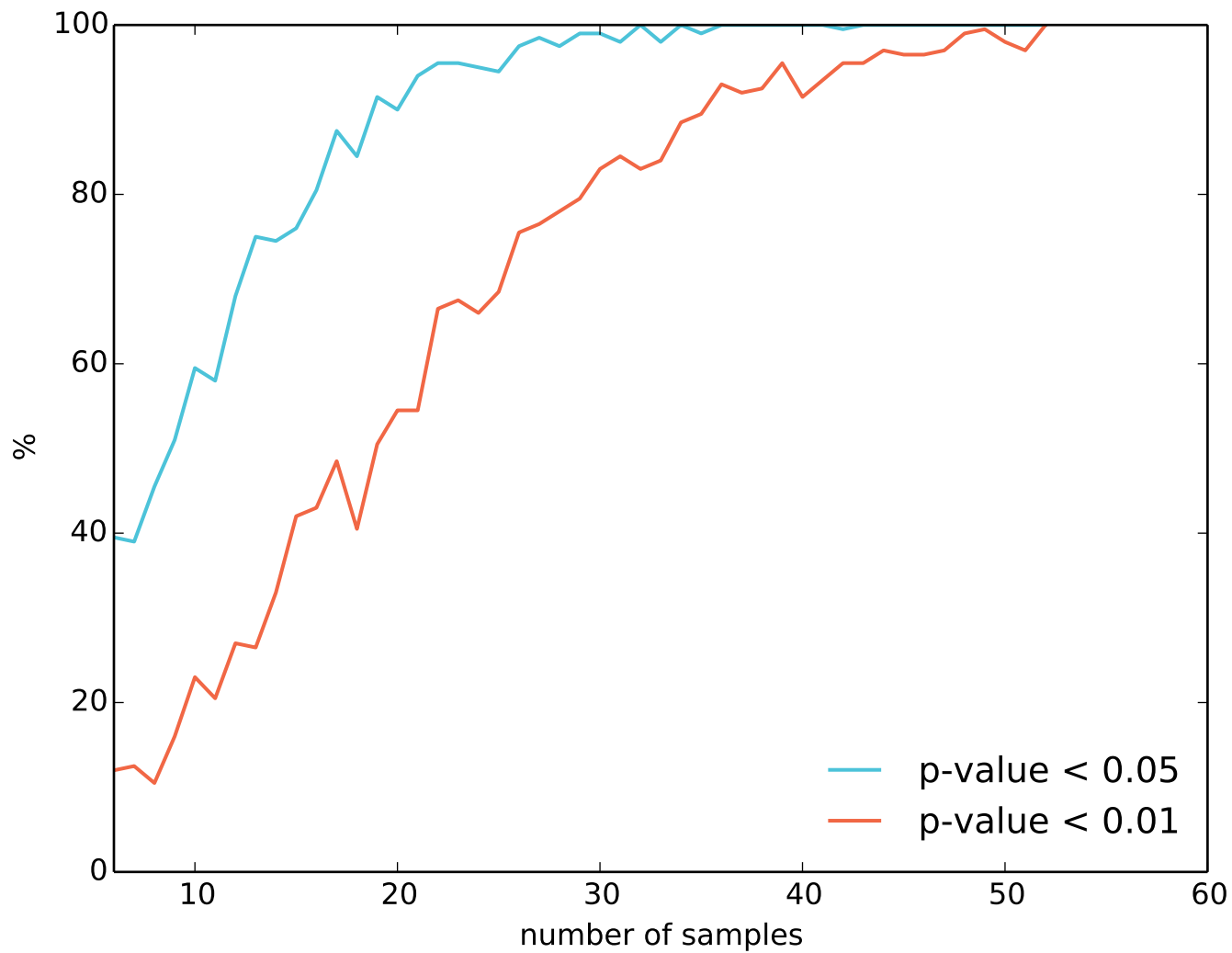

Supplement: Additional file 8: Figure S8. — Percentage of datasets with PCs with a significant correlation (P < 0.05 and P < 0.01) with the age of participants after cell composition was subtracted. The datasets of different sizes were generated with random sampling from a larger cohort. We observed that an age PC can be found with a probability of approximately 60% only in datasets with >22 samples. [file 13072_2015_11_MOESM8_ESM.pdf]

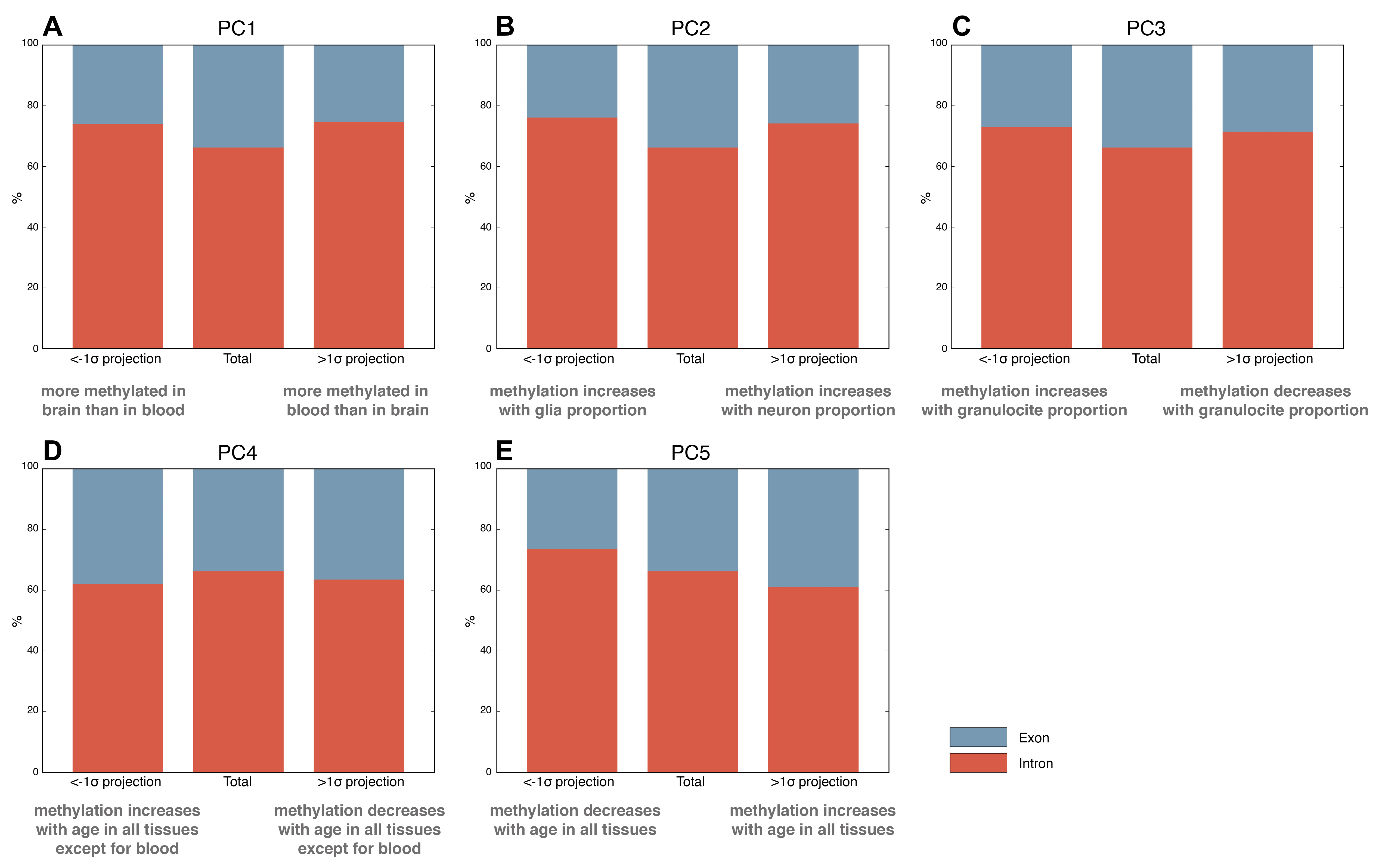

Supplement: Additional file 9: Figure S9. — Enrichment and depletion of intron/exon categories in subsets of CpG sites with < − σ projections (left bar) and > σ projections (right bar) compared to the background total 450K CpG sites (central bar). (A-C) Tissue-related PC1-PC3 showed an enrichment of intron probes and depletion of exon probes irrespective of the sign of the projection. (D) PC4, the age signature found in all tissues except blood showed an enrichment of exon probes and depletion of intron probes in both positive and negative projections. (E) PC5, the age signature that was found present in all of the tissues checked was found enriched in exon probes in positive projections (CpGs increase methylation with age) and enriched in intron probes in negative projections (CpGs decrease methylation with age). PC, principal component. [file 13072_2015_11_MOESM9_ESM.jpeg]

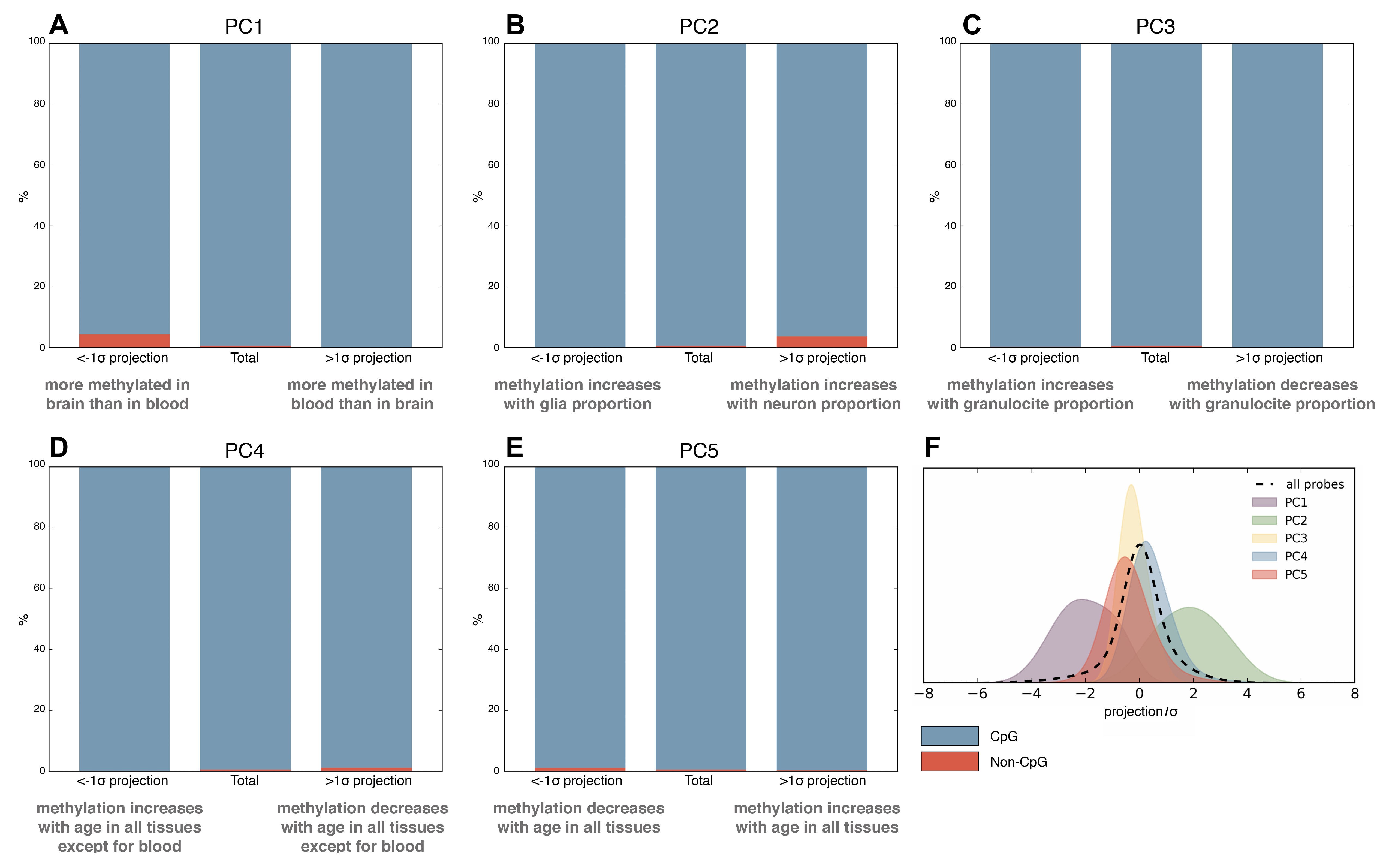

Supplement: Additional file 10: Figure S10. — Enrichment and depletion of CpG/non-CpG categories in subsets of probes with < − σ projections (left bar) and > σ projections (right bar) compared to the background total 450K probes (central bar). PC1 showed an enrichment of non-CpG sites for probes with negative projections (probes more methylated in brain than blood) (A). For PC2, probes with positive projections were enriched for non-CpG (methylation increases with neuron fraction) (B). PC3 showed a general depletion of non-CpG for both projection signs (C). The age-related PCs, PC4 and PC5, showed a slight enrichment of non-CpG sites in the probes where methylation increases with age (positive projections in PC4 and negative in PC5) (D,F). (E) Projections of non-CpG sites in each PC normalized by the standard deviation of all 450K probe projections in the PC. PC, principal component. [file 13072_2015_11_MOESM10_ESM.jpeg]

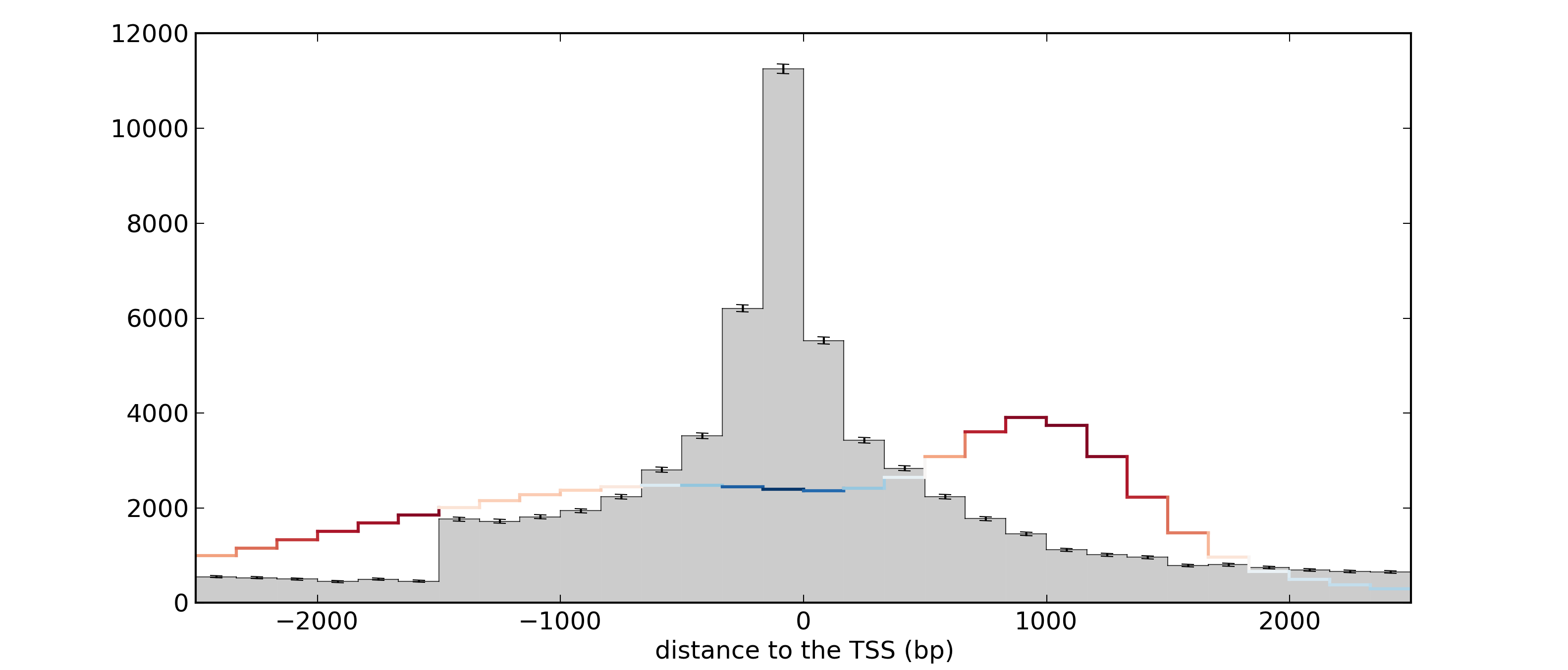

Supplement: Additional file 11: Figure S11. — Scheme of the construction of spatial enrichment heatmaps. In gray we show the background number of probes in each distance bin. Error bars show the standard deviation of a binomial distribution. The color plot is a hypothetical example of an experimental distribution of probes. The number of standard deviations away from the mean background (Z-score) is mapped to a color, where red corresponds to enrichment and blue corresponds to depletion. TSS, transcriptional start site. [file 13072_2015_11_MOESM11_ESM.png]

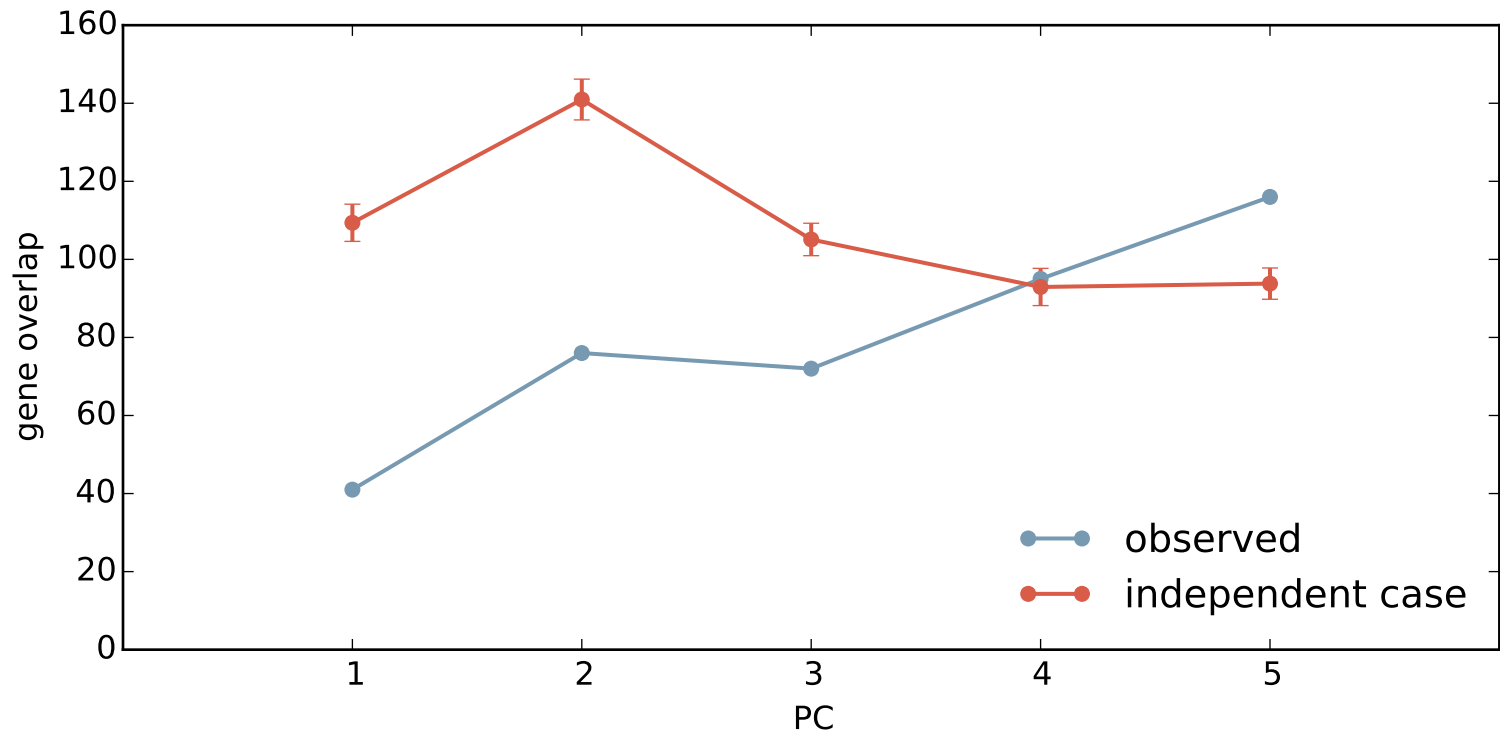

Supplement: Additional file 12: Figure S12. — Overlaps of genes containing probes with positive and negative 2σ projections on the first five PCs. The independent-case overlaps were calculated as the product of the observed probabilities of belonging to each gene set. [file 13072_2015_11_MOESM12_ESM.pdf]

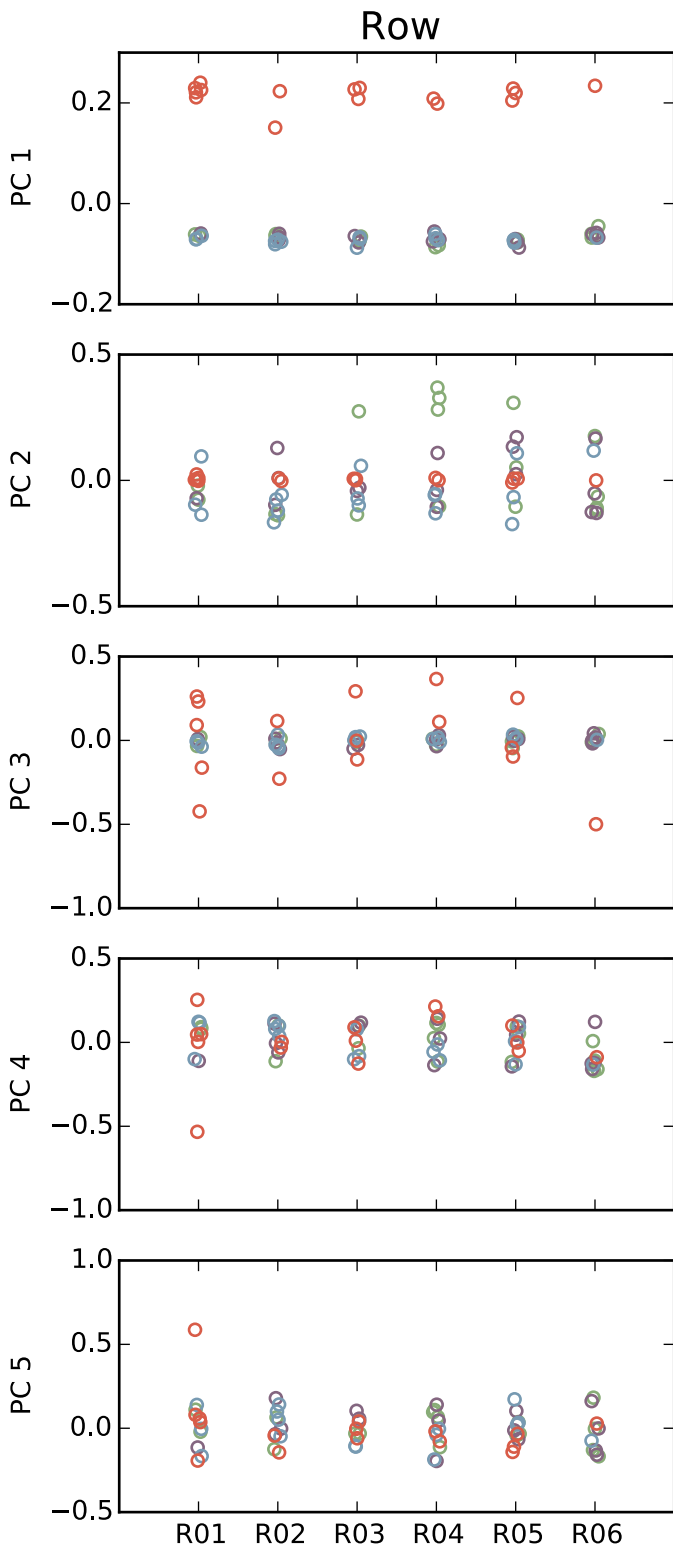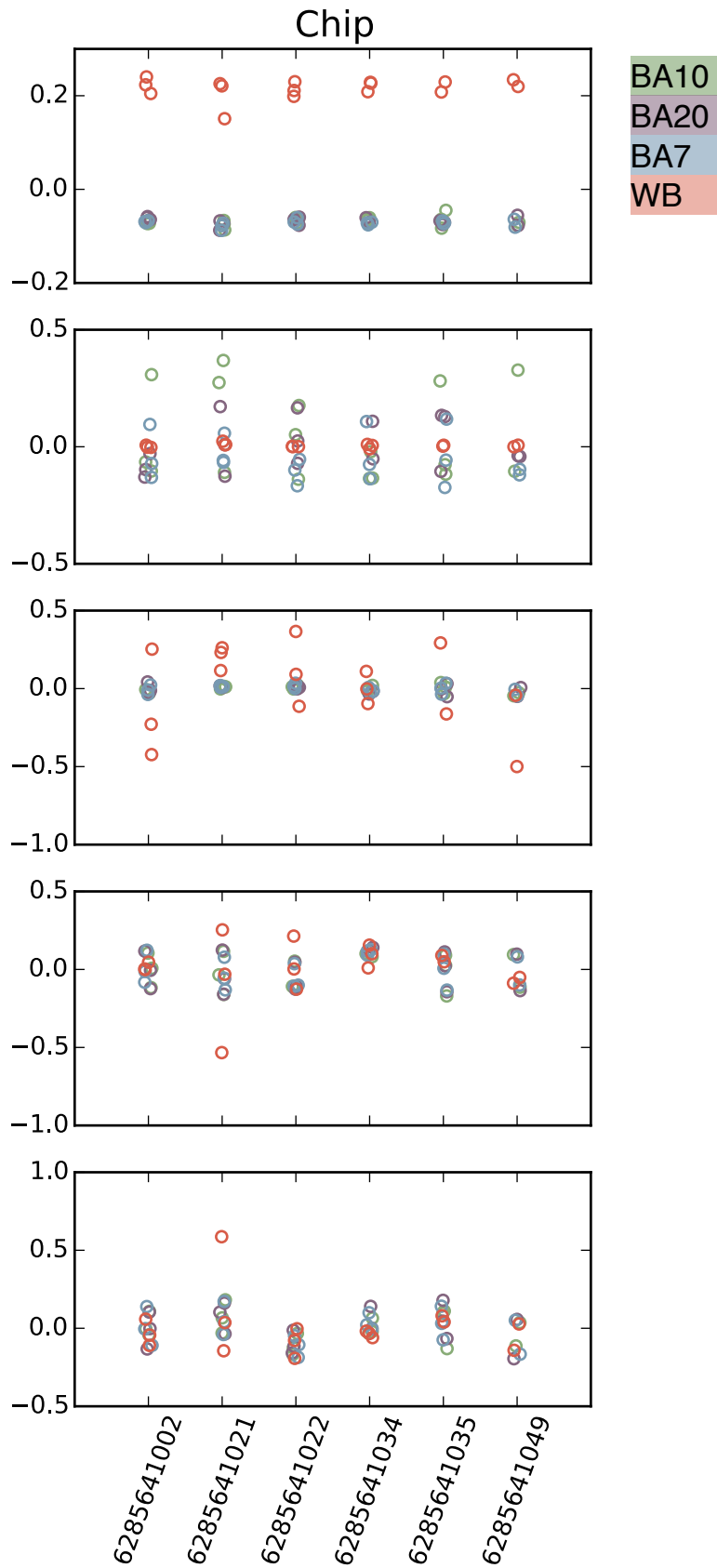

Supplement: Additional file 14: Figure S14. — Distribution of PC scores sorted by chip and row position for the evaluation of possible batch effects. Kolmogorov-Smirnov tests indicated that all distributions were significantly similar. BA10, Broadmann area 10; BA20, Broadmann area 20, BA7, Broadmann area 7; PC, principal component; WB, whole blood. [file 13072_2015_11_MOESM14_ESM.pdf]
