# Supplementary material for: Concordant and discordant DNA methylation signatures of aging in human blood and brain
Source: Epigenetics Chromatin. 2015 May 9;8:19. doi: 10.1186/s13072-015-0011-y (PMC4430927; doi:10.1186/s13072-015-0011-y)
Supplement: Additional file 2: Figure S2. — Scatter plot of PCs vs sample features. PC1 was correlated with tissue of origin. PC2 was correlated with neuron proportion in brain samples. PC3 was correlated with granulocyte proportion of whole blood samples. PC4 was correlated with age in brain samples but not in whole blood samples. PC5 was correlated with age in both brain and whole blood samples. BA10, Broadmann area 10; BA20, Broadmann area 20, BA7, Broadmann area 7; PC, principal component; WB, whole blood. [file 13072_2015_11_MOESM2_ESM.pdf]

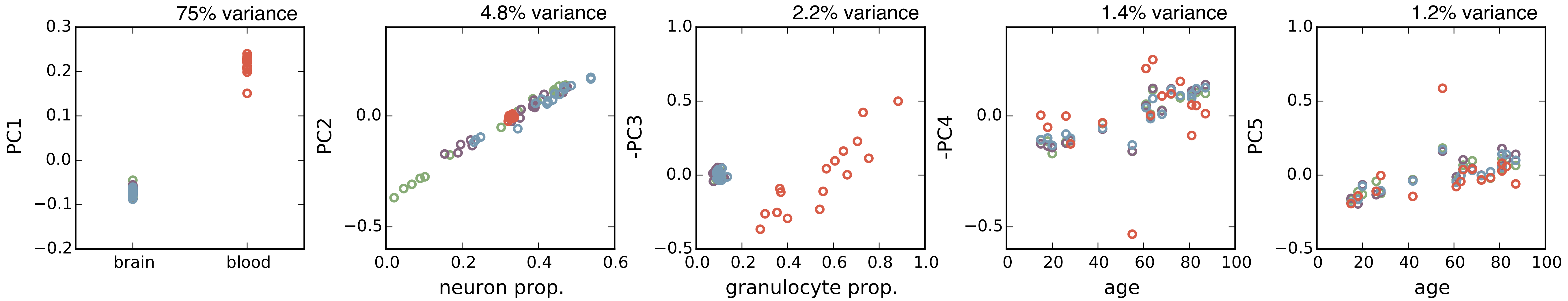

tissue	r, p
ALL	0.99, 9.8e-13

tissue	r, p
BA10	0.97, 1.1e-10
BA20	0.97, 2.3e-10
BA7	0.96, 5.7e-10
WB	0.12, 0.64

tissue	r, p
BA10	0.17, 0.49
BA20	0.23, 0.38
BA7	0.06, 0.81
WB	0.90, 1.7e-6

tissue	r, p
BA10	0.83, 3.8e-5
BA20	0.85, 2.8e-5
BA7	0.91, 4.1e-7
WB	0.40, 0.12

tissue	r, p
BA10	0.73, 8.3e-4
BA20	0.84, 4.6e-5
BA7	0.82, 6.0e-5
WB	0.56, 0.023
