# Supplementary material for: Concordant and discordant DNA methylation signatures of aging in human blood and brain
Source: Epigenetics Chromatin. 2015 May 9;8:19. doi: 10.1186/s13072-015-0011-y (PMC4430927; doi:10.1186/s13072-015-0011-y)
Supplement: Additional file 13: Table S1. — List of GO term enrichment results from DAVID. [file 13072_2015_11_MOESM13_ESM.pdf]

Table S1: List of GO term enrichment results from DAVID.

PC	Direction	Meaning	Score	Terms
1	Positive	More methylated in blood than brain	3.93	neuron projection morphogenesis<br>neuron projection development<br>axonogenesis<br>cell projection morphogenesis<br>cell part morphogenesis<br>cell morphogenesis involved in neuron differentiation<br>cell projection organization<br>neuron development<br>cell morphogenesis<br>cell morphogenesis involved in differentiation<br>cellular component morphogenesis<br>neuron differentiation<br>axon guidance
			1.81	modification-dependent protein catabolic process<br>modification-dependent macromolecule catabolic process<br>macromolecule catabolic process<br>proteolysis involved in cellular protein catabolic process<br>cellular protein catabolic process<br>protein catabolic process<br>cellular macromolecule catabolic process<br>proteolysis
	Negative	More methylated in brain than blood	1.86	defense response<br>inflammatory response<br>response to wounding
			1.44	Rho protein signal transduction<br>Ras protein signal transduction<br>small GTPase mediated signal transduction
2	Positive	More methylated in neurons than glia	None	
	Negative	More methylated in glia than neurons	2.30	induction of programmed cell death<br>induction of apoptosis<br>positive regulation of apoptosis<br>positive regulation of programmed cell death<br>positive regulation of cell death

				regulation of programmed cell death<br>regulation of cell death<br>regulation of apoptosis<br>regulation of Ras protein signal transduction<br>programmed cell death<br>apoptosis<br>regulation of small GTPase mediated signal transduction<br>cell death<br>death<br>regulation of Rho protein signal transduction<br>induction of apoptosis by extracellular signals
			1.74	transmission of nerve impulse<br>synaptic transmission<br>cell-cell signaling<br>neurological system process
			1.38	protein modification by small protein conjugation or removal<br>protein ubiquitination<br>protein modification by small protein conjugation
3	Positive	More methylated in granulocytes than non-granulocytes	2.47	localization of cell<br>cell motility<br>cell migration<br>cell motion
			1.89	inflammatory response<br>response to wounding<br>defense response
			1.46	regulation of cellular localization<br>regulation of secretion<br>positive regulation of transport<br>positive regulation of secretion
	Negative	More methylated in non-granulocytes than granulocytes	2.18	defense response<br>response to wounding<br>inflammatory response
			1.45	regulation of cell activation<br>positive regulation of DNA metabolic process<br>regulation of DNA metabolic process
4	Positive	Decrease DNA methylation with age in all tissues except blood	None	

	Negative	Increase DNA methylation with age in all tissues except blood	3.05	regulation of transcription<br>regulation of RNA metabolic process<br>regulation of transcription, DNA-dependent<br>transcription
			2.39	endocrine system development<br>pancreas development<br>endocrine pancreas development
			2.18	cell fate commitment<br>cell fate specification<br>neuron fate commitment<br>neuron fate specification
			1.52	cell-cell adhesion<br>cell adhesion<br>biological adhesion
			1.42	embryonic organ development<br>chordate embryonic development<br>embryonic development ending in birth or egg hatching<br>in utero embryonic development
			1.40	endocrine system development<br>neuron fate commitment<br>spinal cord development<br>cell differentiation in spinal cord
5	Positive	Increase DNA methylation with age in all tissues	2.03	anterior/posterior pattern formation<br>regionalization<br>pattern specification process
			1.45	morphogenesis of embryonic epithelium<br>embryonic morphogenesis<br>epithelium development<br>embryonic epithelial tube formation<br>tube lumen formation<br>morphogenesis of an epithelium<br>tissue morphogenesis<br>primary neural tube formation<br>neural tube formation<br>neural tube development<br>epithelial tube morphogenesis<br>embryonic development ending in birth or egg hatching

				chordate embryonic development<br>tube morphogenesis<br>tube closure<br>neural tube closure<br>tube development<br>in utero embryonic development
			1.45	endocrine system development<br>endocrine pancreas development<br>pancreas development
			1.36	regulation of transcription<br>regulation of transcription, DNA-dependent<br>regulation of RNA metabolic process<br>transcription<br>positive regulation of gene expression<br>regulation of transcription from RNA polymerase II promoter
	Negative	Decrease DNA methylation with age in all tissues	None	
